# Cardiac-Derived ECM Microspheres for Enhanced hiPSC-CMs Maturation

**DOI:** 10.1002/adfm.202524938

**Published:** 2026-02-22

**Authors:** Jiazhu Xu, Joel Aboagye, Marcella Edwards, Nick Rogozinski, Yufeng Wen, Angello Huerta Gomez, Zui Pan, Ge Zhang, Huaxiao Yang, Yi Hong

**Affiliations:** 1 Department of Bioengineering, University of Texas at Arlington, Arlington, Texas, USA; 2 Department of Biomedical Engineering, University of North Texas, Denton, Texas, USA; 3 Department of Graduate Nursing, University of Texas at Arlington, Arlington, Texas, USA; 4 Department of Biomedical Engineering, The University of Akron, Akron, Ohio, USA

**Keywords:** Decellularized cardiac ECM, hiPSC-CMs, Maturation, Microsphere

## Abstract

The phenotypic immaturity of human induced pluripotent stem cell-derived cardiomyocytes (hiPSC-CMs) remains a critical barrier to their effective use in disease models, drug screening, and cardiac regeneration. Current culture platforms still find it hard to provide a physiologically relevant, stable, and scalable microenvironment to support cardiomyocyte maturation. We report a reproducible heart tissue-derived cardiac extracellular matrix (ECM) microsphere that integrates native biochemical cues with a porous three-dimensional (3D) architecture to support cardiac cell culture and maturation. These microspheres supported the attachment and proliferation of C2C12, HL-1, H9c2, and hiPSC-CMs culture, suggesting the broad applicability of multiple cell types. Compared with the conventional two-dimensional (2D) culture system, decellularized cardiac ECM microspheres provided a spherical 3D culture interface and significantly enhanced hiPSC-CM maturation, as indicated by upregulation of cardiac genes (*ACTA2, TNNT2, GJA1*), rapid calcium cycling, and synchronized calcium transients. Long-term culture (up to 8 months) on these ECM microspheres supported hiPSC-CM maturation, as evidenced by enhanced sarcomere alignment, robust *α*-actinin expression, contractile phenotype, elevated gap junction (connexin 43, CX-43) protein expression, and increased binucleation. This ECM microsphere system represents a scalable and bioactive platform for long-term cardiomyocyte culture and maturation and broader applications in cardiac tissue engineering.

## 1 | Introduction

Human induced pluripotent stem cell-derived cardiomyocytes (hiPSC-CMs) hold great potential for in vitro disease models, drug screening, and cardiac repair due to their ability to provide patient-specific, functional cardiomyocytes at scale [1–7]. However, the generated hiPSC-CMs remain phenotypically immature following differentiation, exhibiting underdeveloped morphology, physiological function, and electrophysiological properties compared to adult CMs [8–10]. This immaturity fundamentally limits the physiological relevance, predictive accuracy, and translational reliability of hiPSC-CM-based cardiac models, representing a major bottleneck for both basic research and clinical translation [11–13]. Despite significant advances in differentiation and stimulation strategies, most existing traditional culture systems fail to recapitulate the complex and dynamic cardiac microenvironment required for sustained cardiomyocyte maturation [10]. Consequently, there remains a critical unmet need for engineered culture platforms that can provide physiologically relevant biochemical and biophysical cues while supporting progressive, long-term structural and functional maturation of hiPSC-CMs in a stable and scalable manner.

Recent efforts have focused on enhancing hiPSC-CM maturation using mechanical and electrical stimuli, metabolic modulation, hormonal cues, co-culture systems, and engineered heart tissues [14–17]. Among these strategies, biomaterial-based 3D platforms have shown considerable promise in enhancing hiPSC-CM maturation by mimicking the native cardiac microenvironment. Unlike conventional two-dimensional (2D) cultures, three-dimensional (3D) systems enable spatial cell–matrix interactions, physiologically relevant mechanical cues, and enhanced cell–cell communication, all of which are critical determinants of cardiomyocyte structural and functional maturation [13]. In particular, microscale materials, such as microparticles or microspheres, provide versatile design parameters, including tunable size, surface chemistry, porosity, and stiffness, which can be precisely engineered to support cell delivery, regulate growth factor release, and advance tissue engineering applications [18]. These properties have made microspheres promising tools for scalable hiPSC expansion and directed cardiac differentiation in bioreactors, as well as for minimally invasive delivery in regenerative medicine [19–28]. Importantly, beyond scalability, microsphere-based and cell-seeded microsphere systems enable several relevant applications, including high-throughput drug screening, suspension-based bioreactor culture, bottom–up tissue assembly, and injectable cell–matrix delivery strategies [18, 29–31]. Accordingly, microparticle- or microsphere-based 3D culture systems have been increasingly explored as modular platforms to overcome the limited physiological relevance and scalability of traditional culture approaches [32, 33]. However, most existing microsphere systems are composed of synthetic polymers or mixed with basic extracellular matrix (ECM) components or fibrin-based ECM-mimetic matrices [21, 33–38], which lack the tissue-specific complexity and bioactivity of the native cardiac ECM, limiting their ability to promote extensive structural and electrophysiological maturation [39, 40].

Accumulating evidence indicates that cardiac-specific ECM cues play an important role in promoting hiPSC-CM maturation. Both cell-derived cardiac ECM and defined cardiac ECM components, including collagen and laminin, as well as fibrin- or Matrigel-based ECM-mimetic matrices, have been used as culture substrates to enhance cardiomyocyte adhesion, sarcomere organization, and functional maturation; however, these approaches typically capture a subset of the biochemical and structural cues present in the native myocardium [38, 41–43]. The cardiac ECM itself is a complex network composed of collagen, elastin, glycosaminoglycans (GAGs), and proteoglycans, organized into a highly structured 3D framework that supports CMs and various non-myocytes, including fibroblasts and endothelial cells in the heart [44]. Notably, matrix components like laminin and Agrin have been shown to facilitate cardiomyocyte maturation [45, 46]. By preserving the native cardiac ECM composition, decellularized cardiac ECM offers a biomimetic microenvironment that closely recapitulates the in vivo niche. For example, decellularized cardiac ECM has been reported to enhance calcium handling and improve the metabolic maturation of hiPSC-CMs [32, 47]. Moreover, decellularized cardiac ECM-based hydrogels derived from porcine myocardium have demonstrated favorable safety and efficacy in preclinical myocardial infarction (MI) models and early-phase clinical trials [48–50].

Building on these findings, cardiac-derived ECM microspheres have emerged as a promising platform that combines the architectural advantages of microscale 3D culture systems with the tissue-specific bioactivity of native myocardium. The spherical geometry provides a high surface-area-to-volume ratio and 3D curvature that promote spatially distributed cell–matrix interactions and support suspension-based culture formats, offering advantages in scalability and long-term culture compared with 2D simple ECM or Matrigel coatings or bulk hydrogels [51]. In contrast to cell-derived ECM or single-component matrices, the use of native cardiac ECM preserves the integrated composition of myocardial-specific proteins, glycosaminoglycans, and matrix-bound signaling factors, offering a physiologically relevant microenvironment that more closely recapitulates the in vivo myocardium niche. This includes mechanical cues such as myocardial-like stiffness, viscoelasticity, and fibrillar architecture that regulate mechanotransduction pathways, including integrin-FAK and YAP/TAZ signaling [52], as well as biochemical cues mediated by preserved ECM components that modulate cardiomyocyte adhesion and maturation [53]. Despite the growing interest in ECM-based microspheres, their application in the context of hiPSC-CMs culture and maturation remains limited. Notably, the use of cardiac ECM microspheres for the structural and functional maturation of hiPSC-CMs has not yet been systematically investigated. Thus, by combining native myocardial matrix cues with a defined 3D microscale architecture, cardiac ECM microspheres present significant potential to advance hiPSC-CM maturation through enhanced cell–matrix interactions and mechanical conditioning.

In this work, we developed the decellularized cardiac extracellular matrix (dcECM) microsphere system to promote the structural and functional maturation of hiPSC-CMs. The microspheres were fabricated using the electrospray technique, and their key properties, including morphology, swelling behavior, water content, and cytocompatibility, were systematically characterized. Different types of cells, such as C2C12, HL-1, and H9c2 cells, were used to evaluate the cytocompatibility of the dcECM microspheres. The response of H9c2 cells to dcECM microspheres was evaluated by assessing cell proliferation and cardiomyogenic differentiation, as an initial measure of the bioactivity and cardiac-specific instructive capacity of the dcECM microspheres. Furthermore, the maturation of hiPSC-CMs was examined over both short- and long-term culture on dcECM microspheres, revealing their potential to serve as a physiologically relevant platform for cardiac tissue engineering.

## 2 | Results and Discussion

### 2.1 | dcECM Microspheres Preparation and Characterization

**FIGURE 1 |:**
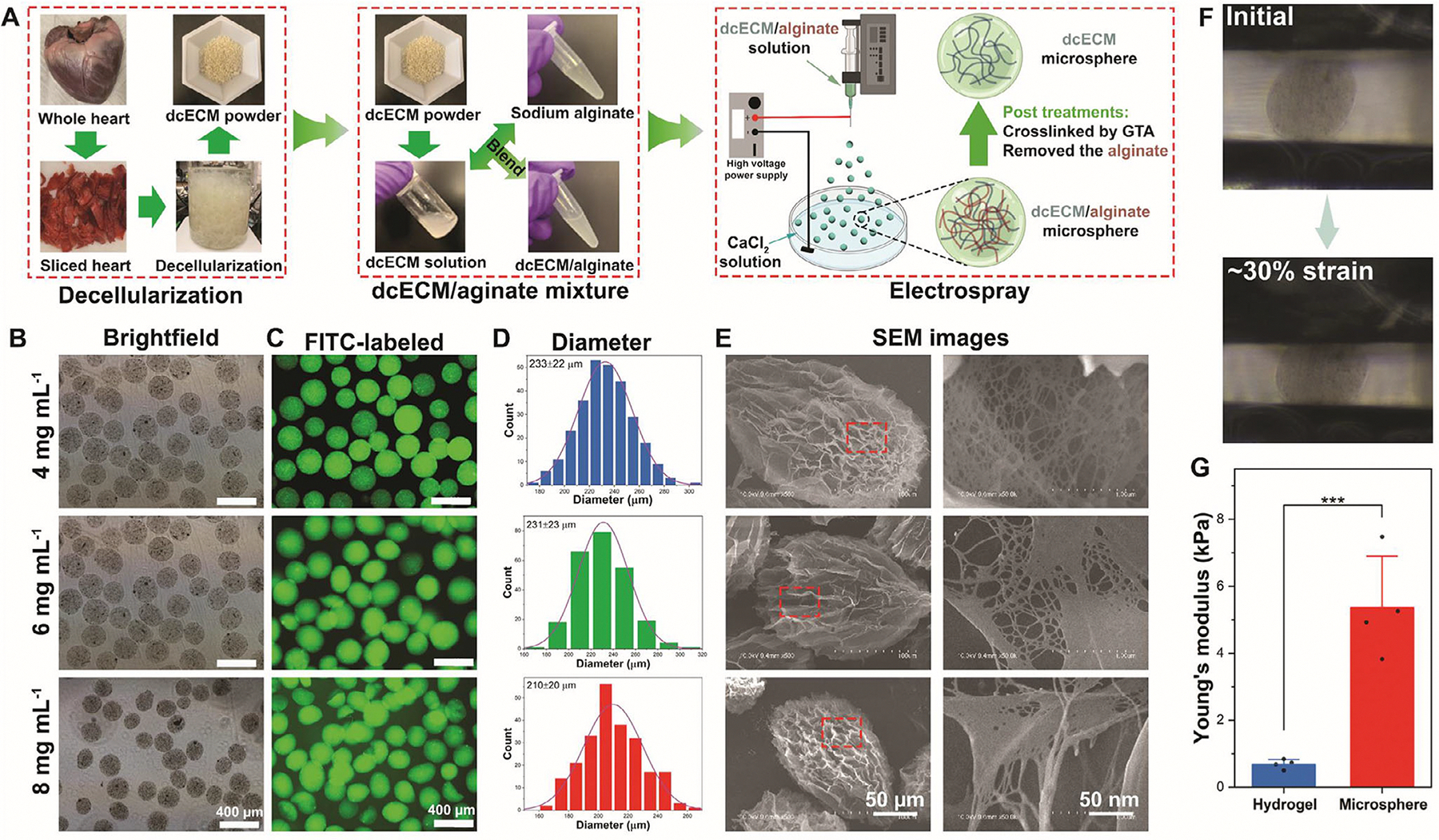
(A) Schematic illustration of dcECM microsphere preparation. (B) Brightfield images of dcECM microspheres (scale bar: 400 μm). (C) Fluorescence images of FITC-labeled dcECM microspheres (scale bar: 400 μm). (D) Diameter distribution of dcECM microspheres (*n* > 200). (E) SEM images of dcECM microspheres. (F) Macroscopic images of the compressive test of the dcECM microsphere with the MicroTester. (G) Young’s modulus of dcECM bulk hydrogel and microsphere (*n* = 4, *** *p* < 0.001). Data are expressed as mean ± SD. Analyzed by a two-tailed Student’s *t-*test.

Porcine cardiac-derived ECM microspheres were fabricated using an electrospray technique. Figure 1A presents a schematic of the electrospray device and the process for dcECM microsphere preparation. Decellularized cardiac ECM was prepared following a previously established protocol [54]. The decellularized cardiac ECM was subsequently digested and neutralized (12 mg mL^−1^), then mixed with sodium alginate (4%) to form a homogeneous precursor solution for microsphere production. The microscale droplets of dcECM/sodium alginate (dcECM/SA) were formed by electrospraying a composite solution of dcECM/SA into a calcium chloride (CaCl_2_, 3% w/v) bath, where immediate ionic crosslinking of alginate enabled rapid microsphere formation. The formed dcECM/SA microspheres were crosslinked with glutaraldehyde (0.5%, v/v). After crosslinking, the alginate was selectively removed using sodium citrate (5%, w/v), and dcECM-based microspheres with a porous structure were obtained. Sodium alginate was reported as a sacrificial crosslinker or stabilizer for scaffold fabrication [55]. dcECM microspheres were prepared using varying concentrations of dcECM solutions to investigate the effect of dcECM content on microsphere morphology, size, and microstructure. Brightfield (Figure 1B) and fluorescence images (Figure 1C) revealed that all groups formed spherical microspheres with relatively uniform morphology after removing the alginate. These microspheres still maintain the spherical shape similar to the dcECM/SA microsphere (Figure S1A). Figure 1D shows the diameter distribution of these microspheres. The diameters of 4, 6, and 8 mg mL^−1^ dcECM microspheres were 233 ± 22 μm, 231 ± 23 μm, and 210 ± 20 μm, respectively. Quantitative analysis of size distributions demonstrated that increasing dcECM concentration led to a decrease in average microsphere diameter. This trend can be attributed to the higher dcECM concentrations may lead to denser crosslinking at the second crosslink with glutaraldehyde, limiting microsphere swelling and resulting in smaller final sizes. The diameter of microspheres was significantly decreased after removing the sodium alginate (Figure S1B). SEM images further revealed the internal architecture of the microspheres (Figure 1E). All dcECM microspheres exhibited an intact and porous surface architecture with a nanofibrous structure after alginate removal. In contrast, the dcECM/SA composite microspheres (Figure S1C) displayed a non-porous surface characterized by groove- and ridge-like topography, resembling the morphology previously reported GelMA-based microspheres [56]. The interconnected ECM microsphere microstructure formed after alginate removal provides a permissive 3D environment that supports extensive cell–matrix interactions and multicellular organization [57]. In addition, microspheres prepared with low dcECM concentration displayed a well-arranged nanofibrous network, whereas those with higher concentrations of microspheres formed coarser, more bundled nanofibers. This transition in fiber morphology likely reflects enhanced molecular entanglement and crosslinking of dcECM proteins at higher concentrations, reinforced by the chemical crosslinking agents [58].

To further evaluate the physicochemical properties of the dcECM microspheres, Fourier-transform infrared (FTIR) spectroscopy, swelling behavior, water content, and compressive mechanical testing were assessed. FTIR spectra (Figure S2A) confirmed the preservation of characteristic dcECM peaks, including amide I (~1650 cm^−1^), amide II (~1550 cm^−1^), and broad O─H/N─H stretching (~3300 cm^−1^), across all dcECM microsphere concentrations (4, 6, and 8 mg mL^−1^).

**FIGURE 2 |:**
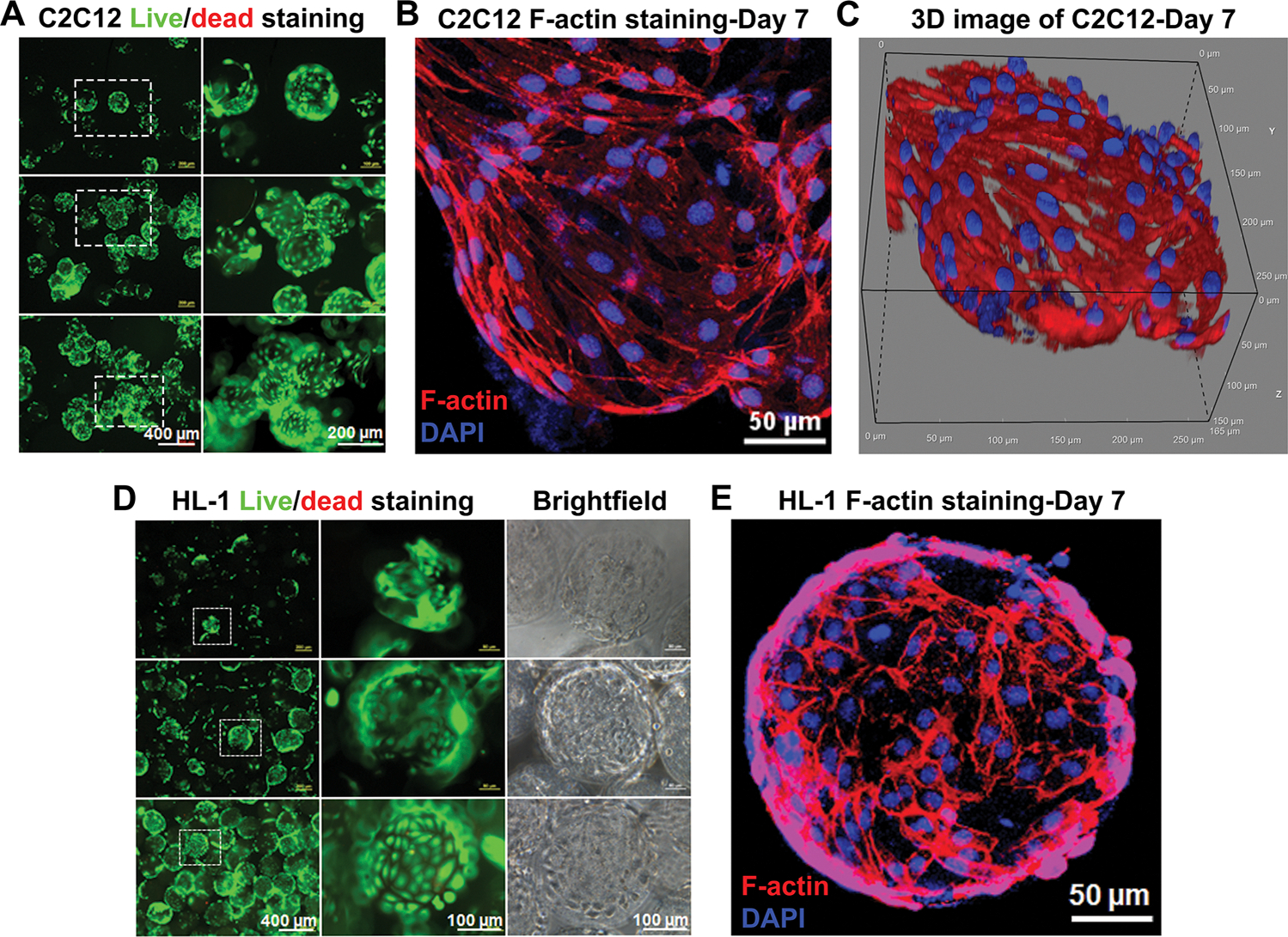
Cellular compatibility of dcECM microspheres seeded with various cell types. (A) Live/dead staining images of C2C12 cell culture on the dcECM microspheres (green: live cells, red: dead cells). (B) The F-actin staining of C2C12 on the dcECM microsphere at day 7. (C) 3D reconstructed confocal image of C2C12 cells on dcECM microspheres at day 7. (D) Live/dead staining and brightfield images of HL-1 cells on dcECM microspheres (green: live cells, red: dead cells). (E) The F-actin staining of HL-1 on the dcECM microsphere at day 7.

Notably, all dcECM microsphere formulations showed similar features to the lyophilized dcECM sponge, indicating that the biochemical composition remained largely intact following microsphere fabrication. Swelling ratio and water content analyses (Figure S2B,C) demonstrated that microspheres retained a high degree of hydration. The swelling ratio increased slightly with dcECM concentration, reaching values over 1300% at 8 mg mL^−1^. Water content also remained high across all groups (>1400%), consistent with the highly porous, hydrated nature of dcECM microspheres. To assess mechanical performance, compressive testing of single microspheres was performed by a MicroTester. Representative images under compression (Figure 2F) showed ~30% strain deformation, confirming the ability of the microspheres to undergo elastic deformation under load. Quantitative measurements revealed that the Young’s modulus of dcECM microspheres (~5.4 kPa) was significantly higher than that of dcECM bulk hydrogel (~0.7 kPa) (Figure 2G), suggesting that microspheres exhibit enhanced stiffness. This is likely due to glutaraldehyde crosslinking and the confined geometry of the microsphere structure [59]. The mechanical strength of substrates plays a critical role in regulating cardiomyocyte behavior, including adhesion, spreading, alignment, and functional maturation [60–62]. Substrates with stiffness approximating native myocardial tissue have been shown to enhance sarcomere organization, promote the expression of cardiac-specific genes and proteins (e.g., *α*-actinin, *TNNT2*), and improve electrophysiological properties such as calcium handling and contraction force [63]. In contrast, excessively soft or overly stiff matrices can impair cell morphology and hinder cardiomyocyte maturation. Crosslinking is an essential strategy for improving the mechanical stability of ECM-based materials; however, its potential impact on ECM bioactivity must be carefully considered [64]. In the present study, crosslinking was introduced specifically to enhance the mechanical strength of cardiac ECM microspheres. While crosslinking may restrict molecular mobility or change the structure of certain ECM components, it does not inherently eliminate cardiac-specific ECM proteins or their bioactive domains [65]. The increase in Young’s modulus achieved through crosslinking provides a mechanically favorable microenvironment that is known to promote cardiomyocyte maturation, including improved sarcomere organization and functional development [10, 66]. Notably, the mechanical properties of the dcECM microspheres were not systematically tuned in this study, representing a limitation and an important direction for future work to decouple mechanical effects from ECM biochemical cues. However, the crosslinking of the dcECM microspheres can primarily serve as a mechanical reinforcement strategy that complements the intrinsic bioactivity of cardiac-derived ECM.

### 2.2 | Cellular Compatibility on dcECM Microspheres

**FIGURE 3 |:**
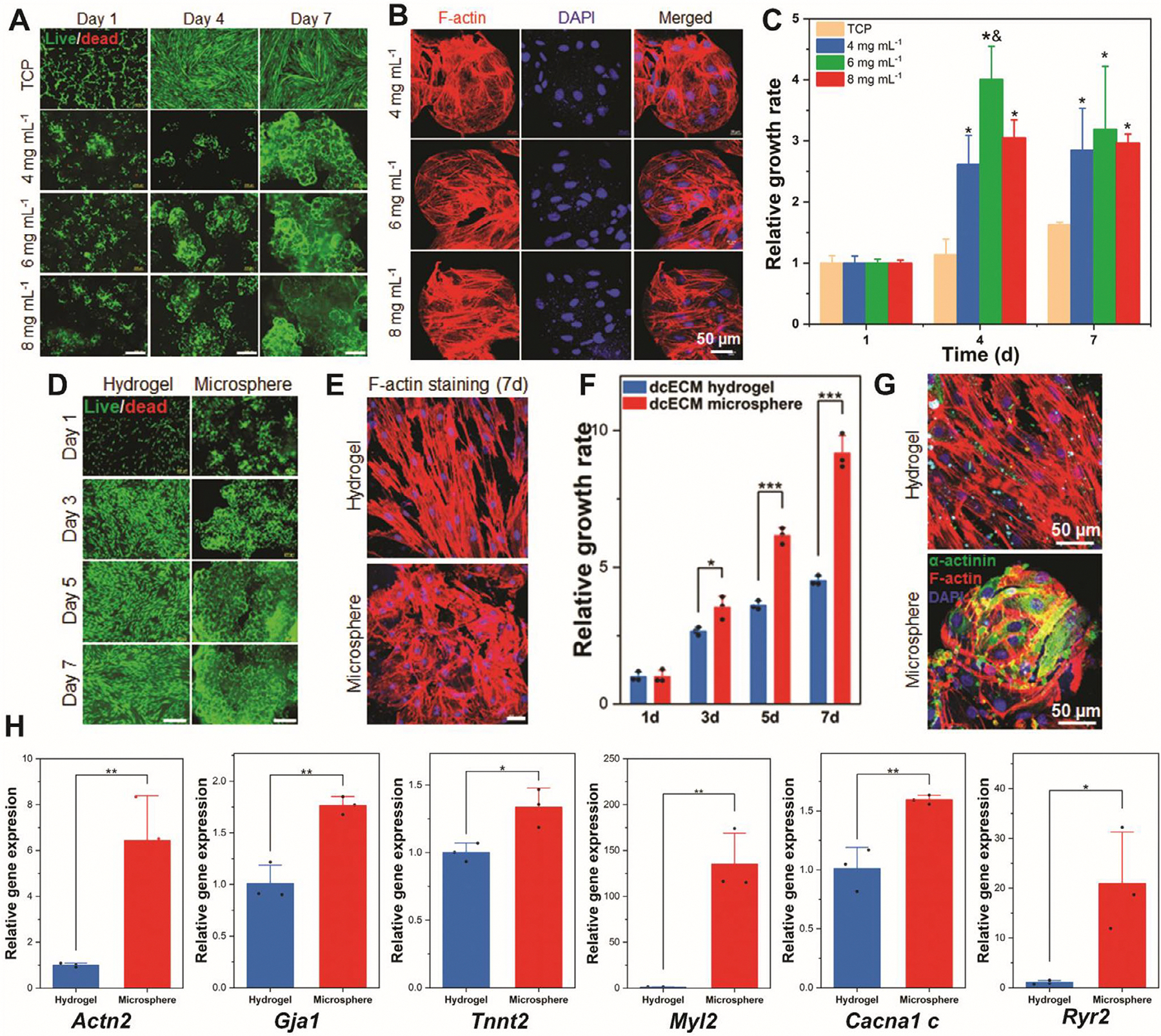
The proliferation and differentiation of H9c2 cells on 2D and 3D culture systems. (A) Live/dead staining images of H9c2 cells on different dcECM microspheres (green: live cells, red: dead cells, scale bar: 400 μm). (B) The F-actin images of H9c2 cells on dcECM microspheres on day 7. (C) The relative cell growth rate of H9c2 cells on different dcECM microspheres and TCP (*n* = 3, * *p* < 0.05 compared with TCP; and *p* < 0.05 compared with 4 mg mL^−1^ dcECM microsphere group, analyzed by One-way ANOVA). (D) Live/dead staining images of H9c2 cells on 2D dcECM bulk hydrogel and 3D microsphere (green: live cells, red: dead cells, scale bar: 400 μm). (E) F-actin staining images of H9c2 cells on 2D dcECM bulk hydrogel and 3D microsphere at day 7 (scale bar: 50 μm). (F) The relative cell growth rate of H9c2 cells on 2D dcECM bulk hydrogel and 3D microsphere (*n* = 3, *p < 0.05, ***p < 0.001, analyzed by two-tailed Student’s *t*-test). (G) Immunofluorescence staining of differentiated H9c2 cells on 2D dcECM bulk hydrogel and 3D microsphere at day 7 (green: *α*-actinin, red: F-actin, blue: DAPI). (H) Gene expression of differentiated H9c2 cells on 2D dcECM bulk hydrogel and 3D microsphere at day 7 (*n* = 3, *p < 0.05, **p < 0.01, analyzed by two-tailed Student’s *t*-test). Data are expressed as mean ± SD.

To evaluate the cytocompatibility and cell-responsive properties of the dcECM microspheres, C2C12 myoblasts and HL-1 cardiomyocytes were cultured on dcECM microspheres and analyzed at various time points (Figure 2). The dcECM microspheres provided a consistent platform to support cell adhesion and maintain phenotypic characteristics across the different lineages evaluated. The C2C12 myoblasts adhered efficiently to the microsphere surface and remained viable throughout the culture period, as shown by live/dead staining. The cells formed multicellular clusters on the microspheres with uniform viability (Figure 2A). Cytoskeletal staining image (Figure 2B) and 3D reconstructed image (Figure 2C) of C2C12 cells further confirmed active spreading and alignment of myoblasts on the microsphere surface. The distribution of C2C12 cells’ orientation angles relative to the poleward direction is shown in Figure S3. The pooled angle distribution (*n* = 125) was strongly skewed toward low angles, with most C2C12 cells oriented within 20° of the poleward direction (Figure S3A). The mean orientation angle was 12.1°, and the alignment order parameter was 0.86, indicating a high degree of directional alignment. The cumulative distribution function (CDF) further quantified the extent of alignment (Figure S3B). Based on a commonly used alignment criterion (*θ* ≤ 15°), 71.2% of C2C12 cells were aligned within 15° of the poleward direction. The defined actin filament organization indicated cytoskeletal remodeling in response to the underlying ECM microtopography. Similarly, the HL-1 cardiomyocytes exhibited high viability on dcECM microspheres over time (Figure 2C). The cells formed interconnected networks, which demonstrated tight integrations with the microsphere surfaces. Brightfield and fluorescent overlay images revealed monolayered structures, and F-actin staining (Figure 2D) showed peripheral cytoskeletal organization without obvious cell alignment. These findings highlight the suitability of the dcECM microspheres to support cardiac cell attachment and growth. Collectively, these results demonstrated that dcECM microspheres can offer a bioinstructive microenvironment capable of supporting the adhesion and proliferation of different cell types. The preservation of structural ECM motifs and native signaling molecules likely contributes to the observed cellular responses.

### 2.3 | Proliferation and Cardiac Differentiation of H9c2 Cells on dcECM Microspheres

The ability of dcECM microspheres to support H9c2 cardiomyocytes’ growth and promote cardiac differentiation was evaluated. As shown in Figure 3A–C, H9c2 cells remained highly viable and proliferative when cultured on dcECM microspheres at various dcECM concentrations (4, 6, and 8 mg mL^−1^) for 7 days. Live/dead staining (Figure 3A) revealed great properties for cell attachment, and widespread cellular viability across all formulations, while phalloidin staining (Figure 3B) confirmed progressive cell spreading and cytoskeletal organization. As the culture time was extended, larger aggregates (Figure 3A) resembling microtissue structures gradually formed, likely due to the ECM secretion of H9c2 cells that bridged and connected adjacent microspheres. Quantification of proliferation (Figure 3C) showed a time-dependent increase in cell growth across all dcECM microsphere groups. Notably, the 6 and 8 mg mL^−1^ microspheres exhibited significantly enhanced proliferation relative to the 2D tissue culture plate (TCP), particularly at days 4 and 7 (*p* < 0.01), suggesting a concentration-dependent improvement in bioactivity and cell support. To further investigate substrate effects, H9c2 cells were cultured on dcECM microspheres versus 2D dcECM hydrogels. Live/dead staining and F-actin organization (Figure 3D,E) confirmed superior cellular coverage and aggregate formation on the microsphere substrates. This observation is aligned with previous findings, where photocrosslinkable methacryloyl platelet lysates (PLMA)-derived microgels supported the adhesion and proliferation of cardiac and endothelial cells, resulting in the formation of cell–microgel aggregates [67]. This highlights a key advantage of microsphere-based scaffolds, namely their capacity to not only support initial cell adhesion and proliferation but also to promote stronger cell–substrate interactions through the formation of cohesive cell–microsphere aggregates. Additionally, the quantitative analysis (Figure 3F) demonstrated a significantly higher relative cell growth rate on dcECM microspheres compared to 2D dcECM hydrogels over the 7-day period, indicating their capacity to support cell proliferation. This enhancement is largely attributed to the unique architectural features of the microspheres, including their high surface-area-to-volume ratio and the presence of interstitial voids between individual spheres [68, 69]. Unlike 2D bulk hydrogels, which often impede cell infiltration until substantial peripheral degradation occurs, microsphere assemblies create a porous microenvironment that facilitates efficient cell migration, proliferation, and spatial colonization [18, 70]. These structural advantages, combined with favorable matrix-derived biochemical cues and mechanical properties, collectively create a more supportive microenvironment for cell attachment, growth, and facilitate tissue-like structure formation.

We also investigated the effect of dcECM concentrations on cardiac differentiation of H9c2 in vitro. H9c2 cells were differentiated on dcECM microspheres after 7 days. Immunofluorescence staining revealed robust expression of sarcomeric *α*-actinin (green) across all groups, with highly organized, striated cytoskeletal structures observed on microspheres with higher ECM content (Figure S4A). CX-43 (red color, Figure S4A), a gap junction protein indicative of electrical coupling, exhibited localized membrane expression and increased density in the 6 and 8 mg mL^−1^ groups, suggesting enhanced intercellular connectivity and syncytial organization. Merged images demonstrate elongated, aligned cell morphologies and densely packed clusters, indicative of advanced cytoskeletal remodeling and multicellular coordination. SEM imaging further supported these observations, showing tight cell–microsphere interaction and extensive surface coverage in all groups (Figure S4B). Gene expression analysis showed no significant differences in *Acta1, Tnnt2*, and *Mlc2v* across dcECM microsphere concentrations, indicating comparable levels of cardiac differentiation (Figure S4C). These results indicated that dcECM microspheres from various dcECM concentrations exhibited a similar cardiac differentiation profile for H9c2 cells.

On the other hand, the influence of 2D versus 3D culture systems on the cardiac differentiation of H9c2 cells was assessed. H9c2 cells cultured on dcECM microspheres (3D) exhibited stronger *α*-actinin expression than those on 2D dcECM bulk hydrogel (Figure 3G). The expressed *α*-actinin spread the entire surface of the microsphere; however, it is barely seen on the 2D dcECM bulk hydrogel group. Furthermore, the gene expression results confirmed that the more mature and differentiated H9c2 cells on the dcECM microsphere (Figure 3H). Quantitative qPCR analysis demonstrated that cardiac-related gene expressions on dcECM microspheres were significantly higher than on 2D dcECM bulk hydrogels. Specifically, the expression levels of *Actn2, Gja1, Tnnt2, Myl2, Cacna1c*, and *Ryr2* were all upregulated on dcECM microspheres, with several genes showing more than a twofold increase. Notably, *Myl2* expression exhibited a dramatic upregulation exceeding 100-fold, indicating a substantial promotion of ventricular-specific maturation [71]. Significantly, the calcium handling-relevant genes, including *Cacna1c* and *Ryr2* [72, 73] were increasingly expressed in 3D dcECM microsphere culture in comparison to the 2D dcECM bulk hydrogel culture. These results suggest that the 3D dcECM microsphere system more effectively supports the cardiac differentiation of H9c2 cells by facilitating improved structural organization, mechanical responsiveness, and functional electrophysiological gene expression compared to conventional bulk hydrogel scaffolds. This is consistent with previous reports showing that ECM-enriched 3D systems, such as fibrin or collagen, enhance cardiomyocyte adhesion, alignment, and maturation [74, 75]. Compared to 2D bulk hydrogel systems, the 3D microsphere offers greater surface area and topographic cues, facilitating cytoskeletal organization and functional gene expression essential for cardiomyogenic commitment.

### 2.4 | Short-Term Functional Maturation of hiPSC-CMs Cultured on dcECM Microspheres

**FIGURE 4 |:**
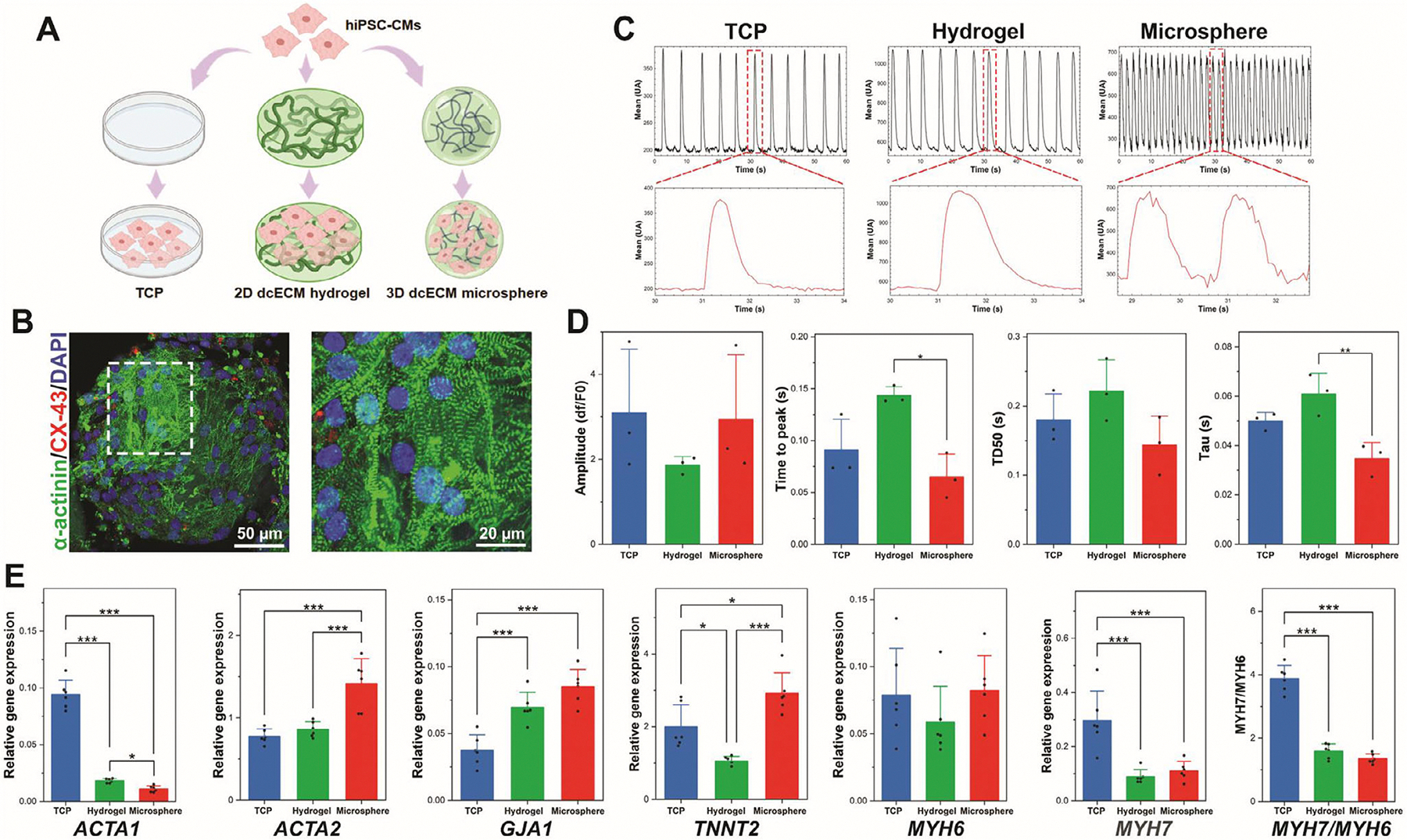
Short-term functional maturation of hiPSC-CMs cultured on dcECM Microspheres. (A) Schematic illustration of hiPSC-CM seeding on different culture platforms: TCP, 2D dcECM hydrogel, and 3D dcECM microspheres. (B) Immunofluorescence staining of hiPSC-CMs on dcECM microsphere at day 14 (green: *α*-actinin, red: CX-43, blue: DAPI, scale bar: 50 μm). The dashed box in the left panel indicates the region shown at higher magnification in the right panel, highlighting the sarcomeric organization (scale bar: 20 μm). (C) Representative calcium transient traces of hiPSC-CMs cultured on TCP, 2D dcECM bulk hydrogel, and dcECM microsphere at day 14. (D) Quantitatively analyze calcium transient from the dynamic plot profiles (*n* = 3, *p < 0.05, **p < 0.01). (E) Gene expression of hiPSC-CMs on TCP, 2D dcECM bulk hydrogel, and dcECM microsphere at day 14 (*n* = 6, *p < 0.05, ***p < 0.001). Data are expressed as mean ± SD. Analyzed by One-way ANOVA.

3D culture systems are increasingly recognized as promising platforms to enhance the structural and functional maturation of hiPSC-CMs [76, 77]. Unlike conventional 2D substrates, which lack physiological architecture and limit cell–matrix interactions, 3D environments provide spatial cues, mechanical resistance, and biochemical signals that more closely resemble native myocardial tissue [3, 10, 78]. To evaluate the ability of dcECM microspheres to support early-stage maturation and functional performance of hiPSC-CMs, cells were cultured for 14 days on dcECM microspheres and compared to 2D dcECM bulk hydrogel and TCP (Figure 4A). Immunofluorescence staining confirmed robust attachment and cytoskeletal development on the microsphere surface (Figure 4B). Cells displayed a highly organized cardiac sarcomere, with aligned *α*-actinin striations and CX-43 expressions, suggesting early formation of gap junctions and structural alignment. To assess excitation–contraction coupling, calcium transient recordings were performed and analyzed for multiple waveform parameters. Representative traces revealed significantly faster calcium kinetics in hiPSC-CMs cultured on dcECM microspheres compared to 2D dcECM bulk hydrogel and TCP controls (Figure 4C). The captured calcium transient videos were shown in Videos S1–S3. The hiPSC-CMs on dcECM microspheres exhibited higher-frequency calcium transients with sharper and more complex waveform profiles. In contrast, the cells on the TCP and 2D dcECM hydrogel groups showed the regular transients, with well-formed peaks and smoother individual waveforms (Figure 4C). These differences indicated that the dcECM microsphere condition is associated with more frequent calcium activity and increased variability in transient shape. Calcium transient analysis revealed distinct functional differences across culture platforms (Figure 4D). hiPSC-CMs on dcECM microspheres exhibited comparable calcium amplitude to TCP, while 2D dcECM bulk hydrogel cultures showed reduced amplitude. Notably, microsphere-cultured cells displayed significantly shortened time-to-peak compared to hydrogel (*p* < 0.05), indicating faster excitation–contraction kinetics. Similarly, both time to 50% decay (TD50) and decay constant (Tau, *p* < 0.01) were lower in the microsphere group, suggesting more rapid calcium release and reuptake. Rapid calcium cycling is a hallmark of mature cardiomyocytes, reflecting efficient excitation–contraction coupling and synchronized electrical activity [10, 79]. These shorter relaxation widths suggest accelerated calcium cycling dynamics, aligning with more mature excitation–contraction coupling [80]. Together, these results demonstrate that the 3D dcECM microsphere system enhances calcium handling dynamics, supporting improved electrophysiological maturation relative to 2D dcECM bulk hydrogel culture.

At the molecular level, gene expression analyses further supported the enhanced maturation phenotype. As shown in Figure 4E, *ACTA1* was significantly downregulated in the dcECM microsphere group compared with the dcECM hydrogel and TCP groups. This downregulation is consistent with cardiomyocyte maturation, as *ACTA1* (*α*-skeletal actin) is progressively repressed in mature CMs [81]. In the meantime, *ACTA2* expression was significantly upregulated (*p* < 0.001), suggesting a shift toward a more mature CM phenotype. The sarcomere gene, *TNNT2*, was significantly up-regulated in the dcECM microsphere group in comparison to the TCP and 2D dcECM hydrogel groups. Furthermore, the electrical coupling marker gene, *GJA1*, was also significantly upregulated in the dcECM microsphere group, suggesting improved electrophysiological function of hiPSC-CMs. However, the *MYH7/MYH6* expression ratio in both the 2D dcECM hydrogel and dcECM microsphere groups was significantly lower than that observed in the TCP group, with no significant difference between these two groups. This outcome may be attributed to differences in substrate stiffness, as the Young’s modulus of the dcECM microspheres (~5.4 kPa, Figure 1G) is substantially lower than that of TCP (~1 GPa), which can influence cardiomyocyte maturation by modulating cytoskeletal organization and the expression of contractile proteins [66, 82]. Additionally, extended culture duration may be necessary for the upregulation of *MYH7*, which typically occurs during later stages of cardiomyocyte maturation. Collectively, these findings demonstrate that the dcECM microsphere platform provides a more biomimetic microenvironment than either 2D dcECM bulk hydrogel or traditional TCP, enhancing electrophysiological function and sarcomere gene maturation of hiPSC-CMs.

### 2.5 | Long-Term Culture and Functional Maturation of hiPSC-CMs on dcECM Microspheres

**FIGURE 5 |:**
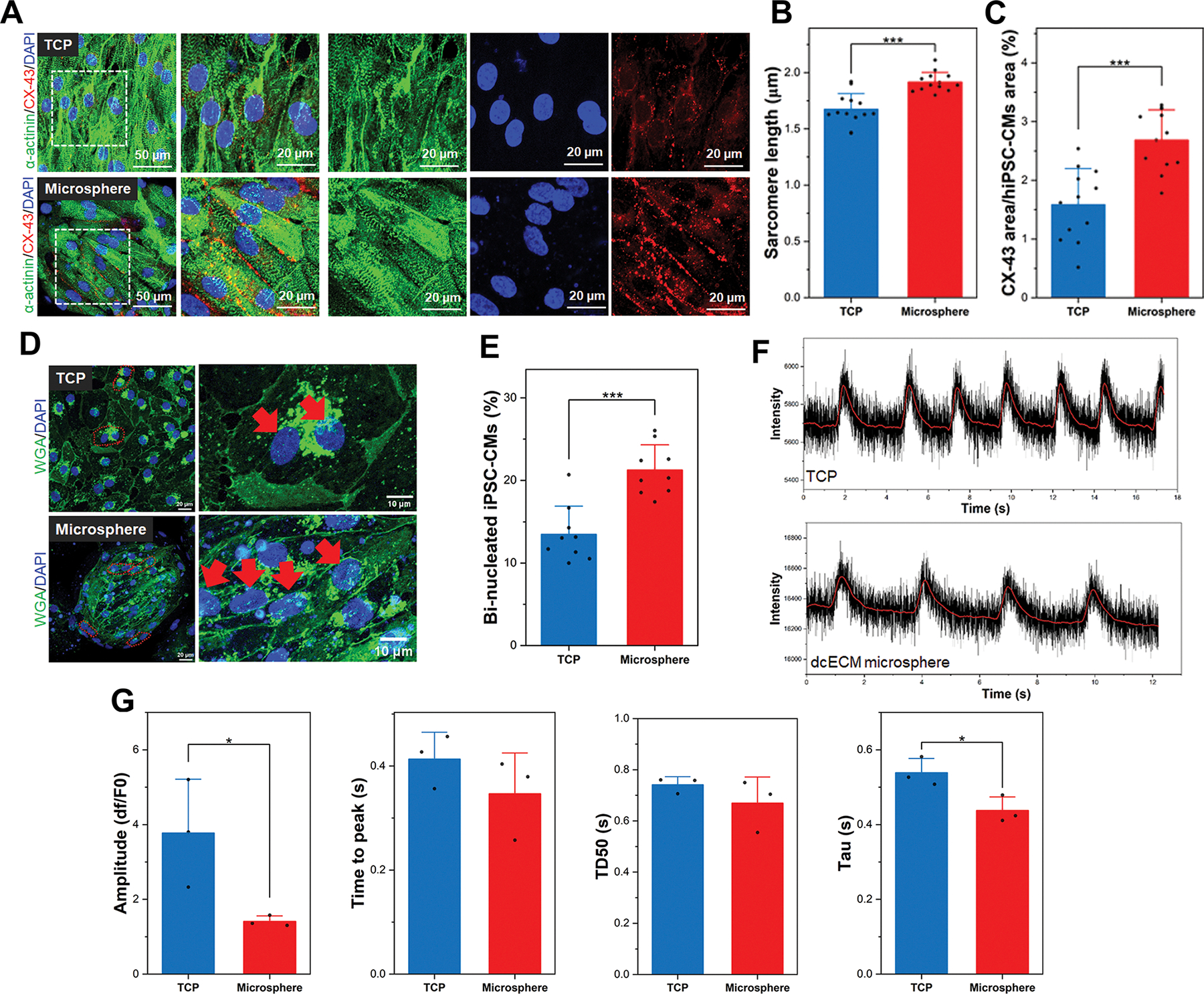
Long-term culture and functional maturation of hiPSC-CMs cultured on dcECM microspheres. (A) Immunofluorescence staining of hiPSC-CMs on TCP and dcECM microsphere at 8 months (green: *α*-actinin, blue: DAPI, and red: CX-43). (B) Quantification of sarcomere length in hiPSC-CMs cultured on dcECM microspheres and TCP (*n* = 12, ***p < 0.001). (C) Quantification of CX-43 area relative to cell area in hiPSC-CMs cultured on dcECM microsphere and TCP (*n* = 12, ***p < 0.001). (D) WGA staining of hiPSC-CMs on dcECM microsphere and TCP (green: cell membrane, blue: DAPI, red arrows: binucleated cells). (E) Quantification of the percentage of binucleated hiPSC-CMs on dcECM microsphere and TCP (*n* = 9, ***p < 0.001). (F) Time-series traces of calcium transient recorded from hiPSC-CMs cultured on dcECM microspheres and TCP for 8 months. The black traces represent raw fluorescence intensity, while the red lines denote smooth transient signals over time. (G) Quantitatively analyze the transient from the dynamic plot profile (*n* = 3, *p < 0.05, ***p < 0.001). Data are expressed as mean ± SD. Analyzed by a two-tailed Student’s *t*-test.

Achieving and maintaining functionally mature hiPSC-CMs is essential for better applications in cardiac regenerative medicine, disease models, and drug screening [10]. While many strategies promote early-stage maturation, sustained maturation is necessary to ensure safety, reliability, and clinical relevance. Immature or partially matured hiPSC-CMs pose risks such as arrhythmogenicity and poor electromechanical integration when implanted, limiting their therapeutic efficacy [14]. Therefore, it is critical to establish culture platforms that not only accelerate early maturation but also support the progressive and sustained development of mature cardiac phenotypes over extended periods. To assess the long-term phenotypic stability and contractile performance, hiPSC-CMs were cultured on dcECM microspheres or TCP for up to 8 months (240 days). Immunofluorescence analysis revealed that hiPSC-CMs maintained well-aligned sarcomere structures and robust expression of *α*-actinin on dcECM microspheres, indicative of advanced structural maturation (Figure 5A). In contrast, TCP-cultured cardiomyocytes showed a more disorganized sarcomere pattern, suggesting that 2D culture conditions limit long-term contractile protein alignment. Furthermore, quantitative measurement of sarcomere length (Figure 5B) demonstrated a significantly greater average sarcomere length in the dcECM microsphere group (1.91 ± 0.08 μm) compared to the TCP group (1.67 ± 0.13 μm), further supporting the enhanced cardiac sarcomere organization and maturation promoted by the 3D microsphere environment. CX-43 staining further revealed significantly higher expression and membrane-localized gap junctions in the microsphere group, suggesting improved intercellular electrical coupling, while the TCP group showed diffuse CX-43 distribution. Quantitative analysis of gap junctions demonstrated a significant increase in CX-43 expression in hiPSC-CMs cultured on dcECM microspheres compared to TCP (Figure 5C). When normalized to the cell area, microsphere-cultured hiPSC-CMs exhibited markedly greater CX-43 coverage, indicative of enhanced cell–cell coupling. These findings suggest that the 3D dcECM microsphere platform promotes the establishment of functional intercellular connections, a critical hallmark of cardiomyocyte maturation. Additionally, wheat germ agglutinin (WGA) staining (Figure 5D) revealed that hiPSC-CMs formed a more binucleated structure (red arrows) in a long-term culture, which was confirmed by the quantitative measure of the number of binucleated hiPSC-CMs (Figure 5E). The microsphere group exhibited a higher percentage of binucleated CMs compared with 2D TCP, indicating a shift toward adult-like CMs [83]. These results demonstrate that prolonged culture over 8 months in a 3D microsphere system promotes advanced structural organization, increased electrical coupling, and enhanced CMs maturation compared to the conventional 2D culture.

Calcium transient analysis was also performed after 8 months of culture to assess cardiac functional performance. Figure 5F represents the time-series traces of the calcium transient. While both groups showed comparable beating frequencies, the microsphere-cultured hiPSC-CMs displayed more uniform and well-defined transient peaks, indicating improved synchronization. Subsequently, quantitative analysis of calcium transients (Figure 5G) revealed that hiPSC-CMs cultured on dcECM microspheres exhibited significantly reduced amplitude compared to TCP, while time-to-peak and time to 50% decay (TD50) remained comparable. Notably, the decay constant (Tau) was significantly shortened in the microsphere group compared to the TCP group (*p* < 0.05), indicating accelerated calcium reuptake despite diminished release capacity. These results suggest that although calcium transient amplitude was attenuated, enhanced intercellular coupling via CX-43 and cardiac sarcomere organization may contribute to improved synchronization and recovery kinetics, supporting the role of 3D dcECM microspheres in promoting coordinated functional maturation of hiPSC-CMs. Long-term ECM microsphere culture system may influence calcium transient amplitude, release dynamics, and calcium retention, indicating that 3D culture alters calcium handling mechanisms in hiPSC-CMs. While calcium release appears more gradual and reduced in 3D culture, faster clearance suggests adaptations in calcium buffering or reuptake efficiency. These alterations suggest that although 3D microsphere platforms support key aspects of structural organization, further enhanced functional maturation of hiPSC-CMs may require additional cues, such as mechanical and electrical stimulation, to better recapitulate the dynamic in vivo cardiac environment [10, 80, 84].

**FIGURE 6 |:**
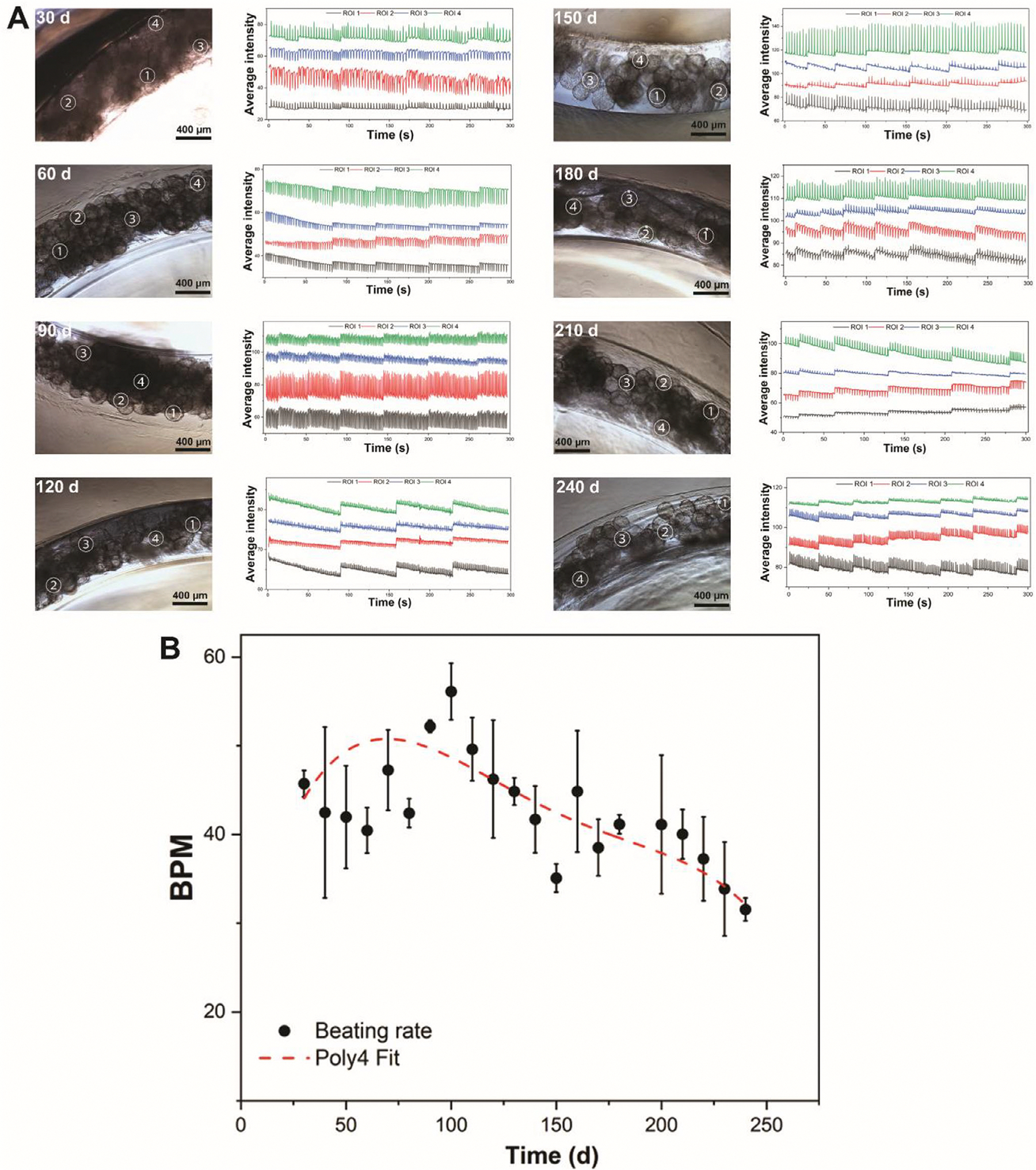
Long-term beating behaviors of hiPSC-CMs on dcECM microspheres (8 months). (A) Beating displacement of hiPSC-CMs on dcECM Microspheres (30 to 240 days). The data includes microscopic images (left, scale bar: 400 μm) showing the hiPSC-CMs/dcECM microsphere and its regions of interest (ROIs) for displacement tracking, and corresponding intensity plots (right) quantifying contraction dynamics over time. (B) Beating rate change of hiPSC-CMs cultured on dcECM microspheres over 240 days (*n* = 5). The black dots represent the beating rate at each time point, while the red dashed line denotes a fourth-order polynomial fitting over time. Data are expressed as mean ± SD.

To evaluate sustained contractile activity of hiPSC-CMs, beating displacement was recorded from day 30 to day 240 using brightfield videos and subsequent motion-tracking analysis in dcECM microsphere-based cultures. Representative images, videos (Videos S4–S11), and corresponding time-dependent displacement traces from multiple regions of interest (ROIs) confirmed rhythmic, synchronized contractions across the microsphere surface over the 8-month period (Figure 6A). In the early stage (30–90 days), hiPSC-CMs exhibited strong, periodic contractions with well-defined displacement amplitudes across all tracked ROIs. As the culture was extended to mid-stage (100–180 days), contractile waveforms of hiPSC-CMs maintained regular frequency and synchronization. The beating rate progressively increased from early culture and reached its maximum around day 100 (Figure 6B), indicating enhanced electrophysiological activity during the initial phase of long-term culture. Between days 100–150, a significant decline in beating frequency was observed, suggesting the onset of functional remodeling. Beyond 150 days, the decrease became more gradual, with cardiomyocytes maintaining measurable contractile activity up to day 240. Polynomial fitting (red dashed line in Figure 6B) further emphasized this trajectory, capturing the peak at ~100 days and the subsequent decline. The reduction in beating frequency over time is consistent with maturation-related adaptations, as more mature cardiomyocytes typically exhibit slower spontaneous beating rates, consistent with adult-like phenotypes [85, 86]. This long-term investigation exhibits the ability of dcECM microspheres to support long-term culture and functional maturation of hiPSC-CMs.

Recent studies have demonstrated that integrating ECM components into 3D culture systems significantly enhances cardiomyocyte maturation by improving metabolic activity, sarcomere structure, and gene expression profiles. For example, decellularized ECM powders incorporated during early cardiac differentiation have been shown to accelerate maturation, increase ventricular marker expression, and enhance calcium handling [32, 47]. Similarly, hydrogel microsphere encapsulation of hiPSCs in suspension culture yielded higher cardiomyocyte yields and improved functional performance, including faster contraction–relaxation kinetics, synchronized calcium transients, and increased β-adrenergic responsiveness [35]. Moreover, biodegradable microcarrier systems have been developed to support both iPSC expansion and cardiac differentiation. These microcarriers preserved cardiac marker expression, protected cells from anoikis and mechanical stress during injection, and enabled functional delivery while maintaining spontaneous contraction and calcium cycling [21]. Building on these strategies, our dcECM-derived microsphere platform combines the structural advantages of 3D microscale architecture with native tissue-specific biochemical cues, promoting early hiPSC-CM maturation, as reflected by upregulation of cardiac-specific genes and improvements in calcium handling dynamics.

The enhanced maturation observed in the 3D dcECM microsphere system is likely driven by the synergistic cell–ECM and cell–cell interactions mediated by biochemical, structural, and mechanical cues. The presence of cardiac-specific ECM components (e.g., collagen, laminin, fibronectin) on the microsphere surface may activate integrin-mediated signaling for further promoting cytoskeletal remodeling, sarcomere organization, and relevant gene expression (Figure 4E) [83, 87–89]. In contrast to conventional 2D substrates, the spherical geometry of microspheres provides an omnidirectional, high–surface-area microenvironment that supports 3D cell–matrix interactions and spatially distributed cell attachment [51]. Unlike flat 2D surfaces, where cells primarily adhere through a single basal interface, spherically curved microscale substrates enable cells to engage ECM cues across multiple spatial axes, resulting in more evenly distributed focal adhesions and force transmission [90]. This geometric support modulates cytoskeletal tension and limits excessive cell spreading and flattening commonly observed on rigid 2D substrates [91], thereby preserving a more physiological cardiomyocyte morphology. Moreover, the surface with spherical curvature promotes localized cell clustering and collective organization by increasing the probability of cell–cell contact along the microsphere surface, thereby facilitating the multicellular formation and enhancing gap junction formation [92] (Figure 5A,D) and further improving electrical coupling. On the other hand, long-term culture of hiPSC-CMs offers significant advantages in promoting extensive maturation across structural, functional, electrophysiological, and metabolic domains, especially in a 3D culture system [93, 94]. Prolonged in vitro maintenance facilitates enhanced sarcomere organization, increased cell size, and binucleation percentage (Figure 5B–E), reflecting morphological features of adult cardiomyocytes. These mature hiPSC-CMs are not only beneficial in vitro but have also demonstrated improved therapeutic outcomes in vivo. Notably, long-term cultured hiPSC-CMs have been shown to promote engraftment, support further in situ maturation, and enhance angiogenesis following transplantation into infarcted rat hearts, underscoring their potential for cardiac regenerative applications [95]. Thus, the combination of cardiac-specific dcECM cues, spherical microscale geometry, and extended culture duration would create a more mimetic microenvironment than 2D culture systems to support hiPSC-CM maturation.

The rationale for the extended culture duration (up to 8 months) is grounded in the protracted timeline of human cardiomyocyte maturation in vivo, which progresses from fetal to postnatal phenotypes over months to years [96]. Consequently, early-stage hiPSC-CMs closely resemble fetal cardiomyocytes, exhibiting immature sarcomere organization, glycolytic metabolism, and underdeveloped calcium handling, whereas prolonged culture under appropriate microenvironmental cues promotes maturation toward late fetal to early postnatal phenotypes, characterized by increased cell size, improved sarcomere alignment, enhanced excitation-contraction coupling, and partial metabolic remodeling [10]. While in vitro maturation is often described using developmental analogs, the precise correspondence between specific hiPSC-CM maturation stages and specific in vivo states (heart development, maturation, and repair) remains incompletely defined. Notably, intermediate to late maturation stages are particularly relevant to cardiac repair, as injured adult myocardium reactivates developmental gene programs and undergoes extensive ECM remodeling, resulting in surviving cardiomyocytes that undergo partial dedifferentiation rather than maintaining a stable adult phenotype [97]. The 8-month culture period in a 3D dcECM microsphere system enables assessment of time-dependent cardiomyocyte maturation under sustained cardiac-specific biochemical and structural cues. It also supports continued structural and functional refinement beyond short-term culture models and provides an in vitro platform to study late-stage maturation and aging-associated cardiomyocyte phenotypes relevant to disease models, drug screening, and regenerative applications.

Despite the significant structural and functional maturation achieved through long-term culture on dcECM microspheres, several limitations remain. While a prolonged culture of hiPSC-CMs supports sarcomere organization, binucleation, and improved CX-43 expression, functional parameters such as calcium transient kinetics, particularly calcium amplitude, did not show significant additional improvement. These discrepancies suggest that functional maturation does not fully progress during the long-term culture and may require additional stimuli such as electrical and mechanical stimulations, or hormonal cues to achieve comprehensive maturation [98]. Furthermore, extended in vitro maintenance has been associated with aging-like phenotypes, including elevated oxidative stress, mitochondrial dysfunction, and activation of senescence-related pathways, which may impair long-term function and contractility [99, 100]. The observed decline in beating rate after 100 days, despite preserved rhythmicity, may reflect such age-associated changes, which will be investigated in future studies focusing on the long-term culture and cardiac aging. In addition, future work incorporating gene expression profiling and RNA sequencing may provide deeper insight into the molecular mechanisms underlying these observations, clarifying whether subpopulations of hiPSC-CMs achieve advanced maturation or display premature aging signatures. These approaches, together with assessments of electrophysiological and metabolic profiling, will enable a more in-depth elucidation of the long-term adaptation of hiPSC-CMs in 3D culture. Overall, these findings highlight the importance of optimizing culture duration and incorporating dynamic stimuli to prevent functional decline and recapitulate the in vivo cardiac microenvironment.

## 3 | Conclusions

We fabricated decellularized cardiac-derived ECM microspheres by the electrospray technique. The size and concentration of the dcECM microsphere are reproducible and tunable through electrospray settings. The dcECM microspheres showed a significantly higher Young’s modulus compared to the 2D dcECM bulk hydrogel. Furthermore, the microspheres promoted adhesion, migration, and proliferation of HL-1, C2C12, and H9c2 cells, and facilitated cardiac differentiation in H9c2 cells. dcECM microspheres enhanced calcium handling and upregulated *ACTA2, GJA1*, and *TNNT2* in hiPSC-CMs during short-term culture. In long-term culture (8 months), dcECM microspheres promoted sustained viability, consistent beating, enhanced *α*-actinin and CX-43 expression, and increased binucleation rates, suggesting advanced structural and electrical maturation of hiPSC-CMs in comparison to the hiPSC-CMs cultured on the TCP. Additionally, hiPSC-CMs maintained consistent contractile activity on dcECM microspheres, with a gradual decline in beating frequency observed after approximately day 100. These results highlight the importance of 3D dcECM microspheres to provide a physiologically relevant microenvironment for hiPSC-CMs maturation and function maintenance. These decellularized cardiac-derived ECM microspheres have shown great promise for further biomedical applications in cardiac repair and regeneration and in vitro disease models in the cardiac field.

## 4 | Experimental Section

### 4.1 | Materials

Sodium dodecyl sulfate (SDS) was acquired from Bio-Rad. Cell counting kit 8 (CCK-8) was purchased from Dojindo. Live and dead viability kit, fetal bovine serum (FBS), fluorescein isothiocyanate (FITC), RPMI 1640 Medium, and B-27 Supplement (50×) were sourced from Thermo Fisher Scientific. Dulbecco’s Modified Eagle Medium (DMEM), Dulbecco’s Phosphate Buffered Saline (1 × DPBS and 10 × DPBS), paraformaldehyde (PFA), agarose, bovine serum albumin (BSA), pepsin, and antibiotic-antimycotic (100×) were obtained from Sigma-Aldrich.

### 4.2 | Preparation of Decellularized Cardiac ECM

Decellularized cardiac ECM (dcECM) was prepared following a previously established protocol [54, 101]. Briefly, fresh porcine myocardium was sliced into sections 1–2 mm sections and treated with 1% (w/v) SDS for 4 days, during which the detergent solution was changed daily. After decellularization, samples were extensively rinsed in deionized (DI) water for 24 h to remove residual SDS. The resulting tissue was then freeze-dried, milled into a fine powder, and stored at −80°C until further use. For solubilization, dcECM powder was digested in 0.01M hydrogen chloride (HCl) containing pepsin (10:1, w/w) under continuous stirring for 24 h at room temperature. The digest was subsequently neutralized on ice to physiological pH (7.4) for further use. To prepare 2D dcECM bulk hydrogels, the neutralized dcECM solution was incubated at 37°C for 30 min to allow gelation.

### 4.3 | Preparation of dcECM Microspheres

dcECM microspheres were fabricated using a customized electrospray apparatus (Figure 1A). Neutralized dcECM solution (12 mg mL^−1^) was thoroughly mixed with sodium alginate (SA) solution to obtain a final dcECM concentration of 4, 6, and 8 mg mL^−1^, while maintaining a constant SA concentration of 2%. The dcECM/SA mixture was loaded into a 20 mL syringe and extruded at a flow rate of 2 mL h^−1^ using a syringe pump through a 25-G blunt needle. The distance between the needle tip and the CaCl_2_ solution was maintained at 7 cm. A high-voltage power supply was used to apply +17.5 kV to the needle and −5 kV to the ground electrode, which was connected via an iron ring to a collection vessel containing 150 mL of 3% calcium chloride (CaCl_2_). Once the electrospray was completed, the obtained dcECM/SA microspheres were filtered with a 100 μm steel sieve and rinsed with DI water thrice to remove the remaining CaCl_2_. Next, dcECM/SA microspheres were crosslinked by 0.5% glutaraldehyde solution overnight in the dark and then rinsed with a large amount of DI water 5 times (30 min/each). After crosslinked, dcECM/SA microspheres were treated with a 5% sodium citrate solution overnight to remove the sodium alginate, followed by three washes with 1 × DPBS (10 min each). For sterilization, the microspheres were immersed in 70% ethanol for 15 min under aseptic conditions within a biosafety cabinet. They were then rinsed five times with DI water (30 min/each) to thoroughly remove any remaining ethanol. Subsequently, the dcECM microspheres were resuspended in 1 × DPBS solution at a concentration of 0.2–0.3 g mL^−1^ and stored at 4°C until further use.

### 4.4 | dcECM Microspheres Characterization

#### 4.4.1 | Size Distribution

The dcECM microspheres were examined using microscopic analysis, and five representative regions were selected for image capture. A minimum of 200 microspheres was subsequently quantified using the ImageJ software. In addition, 10 μg mL^−1^ fluorescein isothiocyanate (FITC) was used to label the dcECM microspheres, the representative images were obtained by a fluorescence microscope (Eclipse Ti-S, Nikon).

### 4.5 | Morphology Analysis

The prepared dcECM microspheres were freeze-dried and subsequently sputter-coated with Au/Pt using a Hummer VI sputtering system. The morphology of the dcECM microspheres was observed using a scanning electron microscope (SEM, Hitachi S-4800).

### 4.6 | H9c2 Proliferation and Differentiation on ECM Microspheres

H9c2 cells (ATCC) were routinely maintained in DMEM containing 10% FBS, 2 mM l-glutamine, 100 μg mL^−1^ streptomycin, and 100 IU mL^−1^ penicillin to support optimal cell growth and maintenance. To establish a non-adhesive culture surface, tissue culture plates (TCP) were pre-coated with a uniform layer of 2% agarose prior to cell seeding. To evaluate cell viability and proliferation of H9c2 on dcECM microspheres, 50 000 cells per 100 μL of dcECM microsphere suspension were seeded into pre-coated 48-well TCP (*n* = 3). Cells were incubated for 2 h at 37°C in a humidified CO_2_ incubator to facilitate initial attachment, after which 500 μL complete culture medium was added. The medium was refreshed every 2 days.

For H9c2 differentiation studies, 100 000 cells per 100 μL of dcECM microsphere suspension were seeded into 24-well TCP (*n* = 3). After a 2-hour incubation at 37°C, 900 μL of complete culture medium was added, and the cells were subsequently cultured for 3 days to reach confluence before being switched to differentiation medium (DMEM containing 2% FBS, 1 μM retinoic acid, 2 mM l-glutamine, 100 IU mL^−1^ penicillin, and 100 μg mL^−1^ streptomycin). During the differentiation process, the medium was replaced every other day, while retinoic acid was replenished daily. The entire culture process was conducted under light-protected conditions.

### 4.7 | hiPSC-CMs Culture on dcECM Microspheres

The Gladstone Institutes generously provided hiPSC lines for use in this study. These hiPSCs were genetically modified using CRISPR-Cas9 technology to stably express the genetically encoded calcium indicator GCaMP [102]. hiPSCs were differentiated into hiPSC-CMs following a previously validated and widely adopted protocol [103]. Briefly, when hiPSCs reached 80%–90% confluency, differentiation was initiated by treatment with 8 μM CHIR99021, a GSK-3 inhibitor and WNT pathway activator, in RPMI 1640/B27 without insulin for 48 h. Following induction, cells were incubated in fresh RPMI 1640/B27 minus insulin for an additional 24 h to allow recovery. Subsequently, WNT signaling was inhibited using 10 μM IWR-1 for 48 h, followed by another 48 h recovery period in RPMI 1640/B27 without insulin. After the recovery phase, the medium was switched to RPMI 1640 containing B27 supplement with insulin, and routine medium changes were conducted every other day. Spontaneous contractions indicative of cardiomyocyte differentiation were typically observed between days 9 and 13. On day 9, purification of hiPSC-CMs was carried out using glucose-free RPMI 1640/B27 with insulin for 48 to 72 h. The resulting purified, beating hiPSC-CMs were then maintained in RPMI 1640 with B27 supplement (with insulin) for subsequent experiments.

For the culture of hiPSC-CMs on dcECM microspheres, hiPSC-CMs were seeded at 100 000 cells per well in 100 μL of dcECM microsphere suspension (~5000 microspheres per 100 μL, and *n* = 3 biological replicates). During the first four days, hiPSC-CMs were cultured in RPMI 1640 + B27 + 4% FBS + antibiotics (100 IU mL^−1^ penicillin, 100 μg mL^−1^ streptomycin). Thereafter, the medium was changed to RPMI 1640 containing B27 and antibiotics without FBS for continued culture.

### 4.8 | Live/Dead Staining and CCK-8 Assay

Live/dead staining (Invitrogen) was performed to assess cell viability. Briefly, samples were washed with 1× DPBS and incubated with 2 μM calcein AM and 4 μM EthD-1 for 30 min at 37°C in the dark, followed by imaging using a fluorescence microscope (Nikon Eclipse Ti-S). CCK-8 assay was used to quantify cell proliferation (*n* = 3). Samples were incubated with a 1:10 CCK-8/DMEM solution for 2 h at 37°C, and absorbance was measured at 450 nm (reference 650 nm) using a microplate reader (Spark 10M, TECAN).

### 4.9 | F-Actin Staining

The cultured cells were fixed with 4% PFA (20 min, room temperature), washed 3 times with 1 × DPBS, and permeabilized with 0.3% Triton X-100 in 1 × DPBS for 10 min. After washing, the cytoskeleton was stained with rhodamine phalloidin (5 μg mL^−1^ in 1% BSA) for 30 min at room temperature. To visualize nuclei, cells were counterstained with 4’,6-diamidino-2-phenylindole (DAPI, Invitrogen). Fluorescence images were obtained using a confocal microscope (Nikon A1R HD25).

### 4.10 | Immunofluorescence Staining and Confocal Imaging

Immunofluorescence staining was performed for target proteins, including *α*-actinin and connexin 43 (CX-43). The samples were fixed with 4% PFA for 20 min at room temperature and permeabilized with 0.3% Triton X-100 in 1 × DPBS for 10 min. To block nonspecific binding, samples were treated with 2% BSA in 1 × DPBS for 1 h at room temperature. Primary antibodies, including anti-*α*-actinin (mouse polyclonal IgG, 1:100, Sigma) and anti-CX-43 (rabbit monoclonal IgG, 1:100, Sigma) were applied in 0.1% BSA and incubated overnight at 4°C. The samples were then washed with 1× DPBS and then incubated with secondary antibodies: goat anti-mouse IgG-488 and goat anti-rabbit IgG-555 (1:200, Invitrogen) for 1 h at room temperature, and nuclei were counterstained with DAPI. Fluorescence images were captured using a confocal microscope (Nikon A1R HD25) with GFP, RFP, and DAPI channels.

### 4.11 | Cell Membrane Staining

For membrane visualization, hiPSC-CMs were stained with Wheat Germ Agglutinin conjugated to Alexa Fluor 488 (WGA, Thermo Fisher Scientific), and nuclei were counterstained with DAPI. Confocal imaging was subsequently performed to capture cellular morphology.

### 4.12 | Quantification of Sarcomere Length, CX-43 Area, and Binucleation

Sarcomere length was quantified from *α*-actinin-stained images acquired using a 40× objective with a confocal microscope (Nikon A1R HD25), using the NIH ImageJ software. CX-43 area was quantified from immunofluorescence images using ImageJ by thresholding and binary segmentation of CX-43 staining, followed by normalization to the corresponding cell area identified from *α*-actinin staining. Binucleation was assessed from WGA-stained images using the same software. A cell was defined as binucleated if two nuclei were fully enclosed within a continuous cell membrane boundary, and the binucleation index was calculated as the percentage of binucleated cells within the analyzed cell population. For each condition, at least nine randomly selected fields were analyzed across three independent samples.

### 4.13 | Calcium Transient Analysis

Calcium transients of genetically edited hiPSC-CMs on the different culture systems can be directly observed under the Nikon A1R HD25 Confocal Microscope. ImageJ software with the Spiky, NDi6d, and Time series analyzer V3 plugins was utilized for data analysis. The Spiky plugin automatically filtered the ROI data to obtain the baseline. Three random ROIs per sample were analyzed. The output graphs were calculated as the change in fluorescent intensity over time, covering 10 s. In addition, calcium handling properties such as amplitude, time to peak, time to 50% decay (TD50), and time decay constant (Tau), were analyzed using MATLAB software to assess the captured video recordings (100 fps, 512 × 512) of hiPSC-CMs (*n* = 3, at least 5 cells per group were quantified from three independent experiments).

In parallel, the IonOptix Calcium and Contractility System (IonOptix, USA) was used to measure the calcium transience of spontaneously contracting GCaMP6f hiPSC-CMs for each condition. Briefly, cells were recorded for about 20 s on an Olympus Microscope IX71 (Olympus, Japan) at 37°C using a ThermoPlate (Tokai Hit, Japan). An excitation wavelength of 470 nm and a FITC filter cube were used to capture intracellular calcium dynamics during the cardiac contraction cycle. Calcium transients were analyzed using the IonWizard Software (IonOptix, USA) to obtain amplitude, time to peak, TD50, and Tau (*n* = 3, a total of 5 cells per condition were analyzed, with 5 transient waves averaged per cell).

### 4.14 | Beating Behavior Analysis of hiPSC-CMs on dcECM Microspheres

At each designated culture time point, spontaneous contractions of hiPSC-CMs on dcECM microspheres were recorded by brightfield microscopy on a temperature-controlled stage. For each sample and time point, short videos were acquired from non-overlapping fields of view (100 fps, ~5 min). At least four non-overlapping, user-defined ROIs were selected and analyzed in MATLAB (MathWorks). For each video, the mean pixel intensity within the fixed ROI was extracted frame-by-frame to generate a one-dimensional contraction trace, which was baseline-corrected and band-pass filtered to suppress drift and high-frequency noise. Beats were identified by peak detection on the processed trace, and beating rate (beats per minute, BPM) was computed from inter-beat intervals and/or total beats over the recording window. Per-video ROI values were summarized as the median to yield a single beating-rate estimate per video for that time point. To visualize the trajectory of beating rate over culture time, a fourth-order polynomial (poly4) least-squares fit was applied to the time-point summaries (least-squares) and plotted as a trend line.

### 4.15 | RT-qPCR

Total RNA was isolated with the RNA Miniprep Kit (Zymo) following the manufacturer’s protocol (*n* ≥ 3). For cDNA synthesis, 4 μL of qScript cDNA SuperMix was mixed with 16 μL RNA solution (100 ng RNA in RNase-free water) and processed in a real-time PCR system (Bio-Rad CFX96) under the following conditions: 25°C for 5 min, 42°C for 30 min, 85°C for 5 min, then held at 4°C. The cDNA products were stored at −20°C until further use. On the other hand, primers for H9c2 (Table S1) and hiPSC-CMs (Table S2) markers were selected based on the previous report [104]. PCR amplification was performed in a 20 μL reaction mixture containing 2 μL cDNA, 1 μL each of forward and reverse primers, 10 μL PerfeCTa SYBR Green FastMix, and RNase-free water. Plates were sealed, centrifuged (3000 *g*, 1 min), and run on the Bio-Rad CFX96 with 40 cycles of 95°C (15 s), 60°C (15 s), and 72°C (45 s), preceded by 1 min at 95°C. Melt curves were generated (60°C–95°C, 0.5°C/s). The 2^*−ΔΔCq*^ method was applied to determine relative gene expression.

### 4.16 | Statistical Analyses

Data are shown as mean ± standard deviation (SD). Comparisons between two groups were evaluated using a two-tailed Student’s *t*-test, whereas multiple group comparisons were assessed by one-way ANOVA with Tukey’s post hoc analysis. Statistical significance was set at *p* < 0.05. Analyses were conducted using Origin software (OriginLab).

## Supplementary Material

Supplementary

Video_S1

Video_S2

Video_S3

Video_S4

Video_S5

Video_S6

Video_S7

Video_S8

Video_S9

Video_S10

Video_S11

Supporting Information

Additional supporting information can be found online in the Supporting Information section.

SupportingFile:adfm74574-sup-0001-SuppMat.docx.

## Data Availability

The data that support the findings of this study are available from the corresponding author upon reasonable request.
