# Supplementary material for: Cardiac-Derived ECM Microspheres for Enhanced hiPSC-CMs Maturation
Source: Adv Funct Mater. Author manuscript; Available in PMC 2026 Jun 2. (PMC13225862; doi:10.1002/adfm.202524938)
Supplement: Supplementary [file NIHMS2171206-supplement-Supplementary.docx]

Supporting Information

**Cardiac-derived ECM Microspheres for Enhanced iPSC-CMs Maturation**

*Jiazhu Xu^1^, Joel Aboagye^2^, Marcella Edwards^2^, Nick Rogozinski^2^, Yufeng Wen^1^, Angello Huerta Gomez^2^, Zui Pan^3^, Ge Zhang^4^, Huaxiao Yang^2*^, Yi Hong^1*^*

1. Department of Bioengineering, University of Texas at Arlington, Arlington, TX 76019

2. Department of Biomedical Engineering, University of North Texas, Denton, TX 76207

3. Department of Graduate Nursing, University of Texas at Arlington, Arlington, TX 76010

4. Department of Biomedical Engineering, The University of Akron, Akron, OH 44325

*Corresponding authors: Yi Hong, [yihong@uta.edu](mailto:yihong@uta.edu), Huaxiao Yang, [huaxiao.yang@unt.edu](mailto:huaxiao.yang@unt.edu)

**1 Characterizations**

**ATR-FTIR**

ATR-FTIR analysis was conducted using a ThermoFisher (Nicolet 6700 FTIR) spectrometer to characterize dcECM microspheres at varying concentrations and the lyophilized dcECM sponge. FTIR spectra were collected over a wavelength range of 400–4000 cm⁻¹, and characteristic peaks were identified to facilitate comparative analysis between the microspheres and the lyophilized dcECM sponge.

**Swelling Ratio**

Lyophilized dcECM microspheres were incubated in 1× DPBS at 37 °C for 24 hours (n = 3). Swelling degree (SD) was determined using **Equation S1**, with *W_S_* and *W_D_* corresponding to the swollen and dry weights of the microspheres, respectively.

***SD* =** $\frac{\boldsymbol{W}_{\boldsymbol{s}}\boldsymbol{-}\boldsymbol{W}_{\boldsymbol{D}}}{\boldsymbol{W}_{\boldsymbol{D}}}\boldsymbol{\times100\%}$ 1

**Water Content**

The water content was calculated using **Equation S2**, where W_1_ refers to the weight of wet dcECM microspheres, and W_2_ indicates the weight of dry lyophilized dcECM microspheres (n = 3).

$\boldsymbol{Water content =}\frac{\boldsymbol{W}_{\boldsymbol{1}}\boldsymbol{-}\boldsymbol{W}_{\boldsymbol{2}}}{\boldsymbol{W}_{\boldsymbol{2}}}\boldsymbol{\times100}$ 2

**Mechanical Strength**

Compressive mechanical testing of 2D dcECM bulk hydrogels was performed using an MTS electromechanical testing system. The dcECM bulk hydrogel precursor solutions were cast into a cylinder mold (4 mm height, 9 mm diameter) and form hydrogels under 37°C for 30 min. Hydrogels (n = 3) were compressed at 1 mm min^-1^ using a 10 N load cell until failure. Young’s modulus was calculated from the linear region (0–10% strain) of the stress–strain curve. The mechanical properties of dcECM microspheres were measured using a micro-scale compression device MicroTester G2 (CellScale biomaterials testing, Canada). Briefly, the samples were submerged in PBS-filled chamber at room temperature and compressed between two parallel plates (microbeam attached to actuator and anvil) for 30 secs at 25% deflection of microsphere diameter. The resulted force (µN) and displacement (µm) values were used to create stress-strain curve and the Young’s modulus was calculated from the following **formulas**:

$\boldsymbol{\emptyset=}\mathbf{cos}^{\boldsymbol{-1}} \boldsymbol{[}\frac{\boldsymbol{R-\delta}}{\boldsymbol{R}}\boldsymbol{]}$ 1

$\boldsymbol{\alpha=(R-\delta)}\tan\boldsymbol{\emptyset}$ 2

$\boldsymbol{f}\left( \boldsymbol{\alpha} \right)\boldsymbol{=}\frac{\boldsymbol{2}\left( \boldsymbol{1+}\boldsymbol{\nu}^{\boldsymbol{2}} \right)\boldsymbol{R}^{\boldsymbol{2}}}{\left( \boldsymbol{\alpha}^{\boldsymbol{2}}\boldsymbol{+4}\boldsymbol{R}^{\boldsymbol{2}} \right)^{\boldsymbol{3}/\boldsymbol{2}}}\boldsymbol{+}\frac{\boldsymbol{1-}\boldsymbol{\nu}^{\boldsymbol{2}}}{\left( \boldsymbol{\alpha}^{\boldsymbol{2}}\boldsymbol{+4}\boldsymbol{R}^{\boldsymbol{2}} \right)^{\boldsymbol{1}/\boldsymbol{2}}}$ 3

$\boldsymbol{E=}\frac{\boldsymbol{3}\left( \boldsymbol{1-}\boldsymbol{\nu}^{\boldsymbol{2}} \right)\boldsymbol{F}}{\boldsymbol{4}\boldsymbol{\delta\alpha}}\boldsymbol{-}\frac{\boldsymbol{f}\left( \boldsymbol{\alpha} \right)\boldsymbol{F}}{\boldsymbol{\pi\delta}}$ 4

F: applied force, R: microsphere radius, δ: displacement, ν: Poisson’s ratio (0.5), E: Young’s modulus

**Cytocompatibility Evaluation with Different Types of Cells**

C2C12 (ATCC) and HL-1were used to investigate the impact microspheres have on cell behavior. C2C12 cells were routinely maintained in DMEM containing 10% fetal bovine serum (FBS), 2 mM L-glutamine, 100 µg mL^-1^ streptomycin, and 100 IU mL^-1^ penicillin to support optimal cell growth and maintenance.

HL-1 cells were cultured on 0.02% gelatin/10 µg mL^-1^ fibronectin-coated surfaces in Claycomb medium (Sigma) supplemented with 10% FBS, 0.1 mM norepinephrine, 2 mM glutamine, 100 µg mL^-1^ streptomycin, and 100 IU mL^-1^ penicillin.

To evaluate cell viability and proliferation of C2C12 and HL-1 on dcECM microspheres, 50,000 cells per 100 µL of dcECM microsphere suspension were seeded into pre-coated 48-well TCP. The cells were incubated for 2 h at 37 °C in a humidified CO₂ incubator to facilitate initial attachment, followed by the addition of 500 µL complete culture medium. The medium was refreshed every 2 days.

**Cell Morphology**

The cultured cells on the dcECM microspheres were fixed in 2.5% glutaraldehyde overnight and washed twice with 1×DPBS. The samples were dehydrated with a gradient ethanol solution (50%, 70%, 80%, 90%, 95%,100%) for 5 minutes at each concentration, with 100% ethanol applied twice. After dehydration, the cells-microspheres constructs were left to air dry before SEM analysis.

**C2C12 Orientation and Alignment Analysis**

C2C12 orientation on dcECM microspheres was quantified from confocal fluorescence images of F-actin–stained cells. For each identified microsphere within an image, one north pole and one south pole were manually marked to define the projected circle center and radius. For each cell, two loci (head and tail) were manually marked to define the cell axis on the hemispherical surface. The axial angle towards the poleward direction of each cell was subsequently computed and summarized using MATLAB. A total of 125 cells from multiple microspheres (n=22) were analyzed. Orientation angles were pooled across samples, and their distribution was visualized as histograms with a bin width of 5**°**, overlaid with a kernel density estimation curve.

Cell alignment was quantified using an alignment order parameter (S), calculated as:

$\boldsymbol{S}=\left\langle\cos\left( 2\theta\right) \right\rangle$ *5*where θ is the axial orientation angle between the cell tangent direction and the local poleward direction, constrained to the range 0–90°.

Cells were classified as aligned if their axial orientation angle (θ), defined relative to the poleward direction, was ≤ 15°. The cumulative distribution function (CDF) of orientation angles was computed by sorting individual angles in ascending order and calculating the cumulative fraction of cells with angles less than or equal to a given value. The percentage of aligned cells was defined as the cumulative fraction at θ = 15°. Three-dimensional (3D) reconstructed images were generated from confocal z-stacks to visualize the spatial organization of cells on the microsphere surface.

***Table S1*** *Primer sequence of H9c2 used for qPCR.*

| Gene | Forward primer | Reverse primer | |
| --- | --- | --- | --- |
| *GAPDH*  *Acta1*  *Tnnt2*  *Mlc2v*  *Myl2*  *Gja1*  *Cacna1c*  *Ryr2*  *Actn2* | ATGGTGAAGGTCGGTGTAA  CACGGCATTATCACCAACTG  GCGGAAGAGTGGGAAGAGACA  CTAAGGGACACGTTTGCTGC  AGGCCTTCACAATCATGGAC  TTGCTGCTGGACATGAACTC  AGCTCAAATTCACTGCCAAGC  CCCACCTCCTTGACATCG  TATTGGGGCTGAAGAAATCG | | TCCACAGTCTTCTGAGTGGC  CCGGAGGCATAGAGAGACAG  CCACAGCTCCTTGGCCTTCT  TTGGACCTGGAGCCTCTTTG  TGGAGAACCTCTCTGCTTGC  ACGGGAACGAAAATGAACAC  ACAATGTCTGTGGCCACGTT  CAGCCAACAAGCCAACAG  CACATTTCGGTATGGTGCTG |

***Table S2*** *Primer sequence of hiPSC-CMs used for qPCR.*

| Primers | | Forward | Reverse |
| --- | --- | --- | --- |
| *ACTA1* | AGGTCATCACCATCGGCAACGA | | GCTGTTGTAGGTGGTCTCGTGA |
| *ACTA2* | CTATGCCTCTGGACGCACAACT | | CAGATCCAGACGCATGATGGCA |
| *GJA1* | GGAGATGAGCAGTCTGCCTTTC | | TGAGCCAGGTACAAGAGTGTGG |
| *TNNT2* | TTCACCAAAGATCTGCTCCTCGCT | | TTATTACTGGTGTGGAGTGGGTGTGG |
| *MYH6* | TCTCCGACAACGCCTATCAGTAC | | GTCACCTATGGCTGCAATGCT |
| *MYH7* | GGCAAGACAGTGACCGTGAAG | | CGTAGCGATCCTTGAGGTTGTA |
| *GAPDH* | GGAGCGAGATCCCTCCAAAAT | | GGCTGTTGTCATACTTCTCATGG |

**2 Supplementary Results**


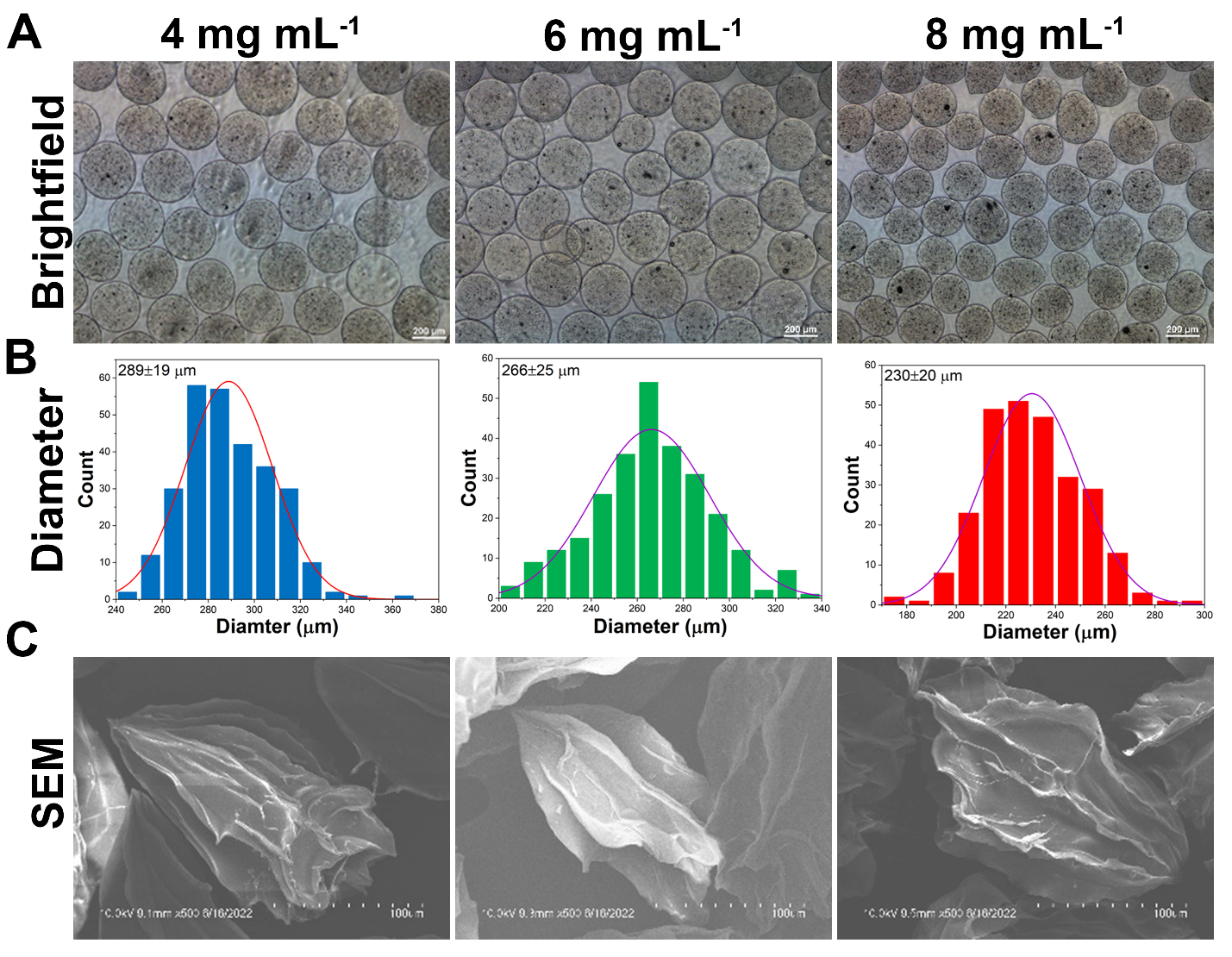


**Figure S1** Characterization of dcECM/Alg microspheres. (A) Brightfield images of different dcECM/SA microspheres (scale bar: 200 µm). (B) Diameter distribution of different dcECM/SA microspheres (n >200). (C) SEM images of different dcECM/SA microspheres (scale bar: 100 µm).


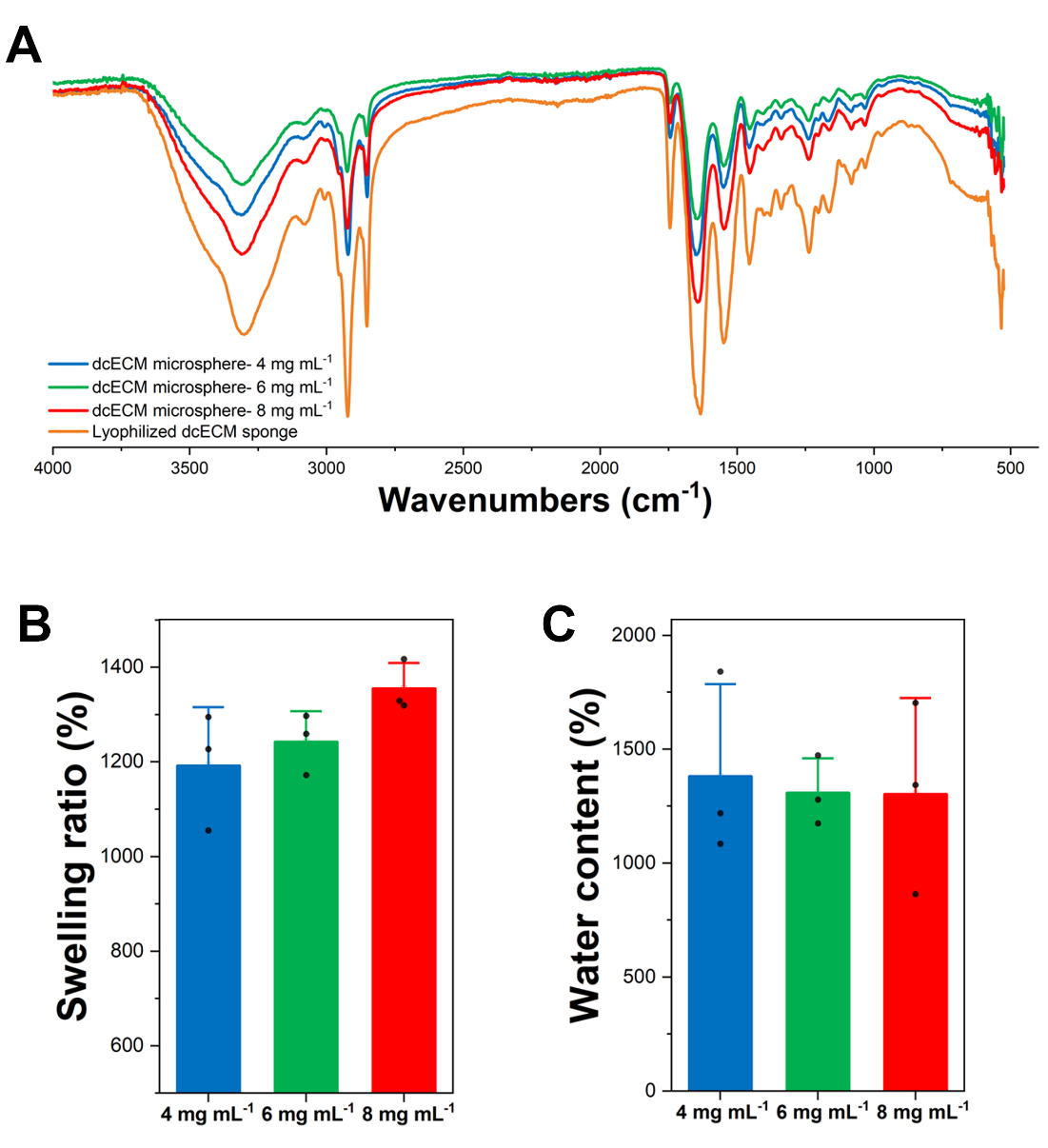


**Figure S2** Characterization of dcECM microspheres. (A) FTIR spectrum of dcECM sponge and different dcECM microspheres. (B) Swelling ration of dcECM microspheres (n=3). (C) Water content of dcECM microspheres (n=3). Data are expressed as mean ± SD. Analyzed by One-way ANOVA.


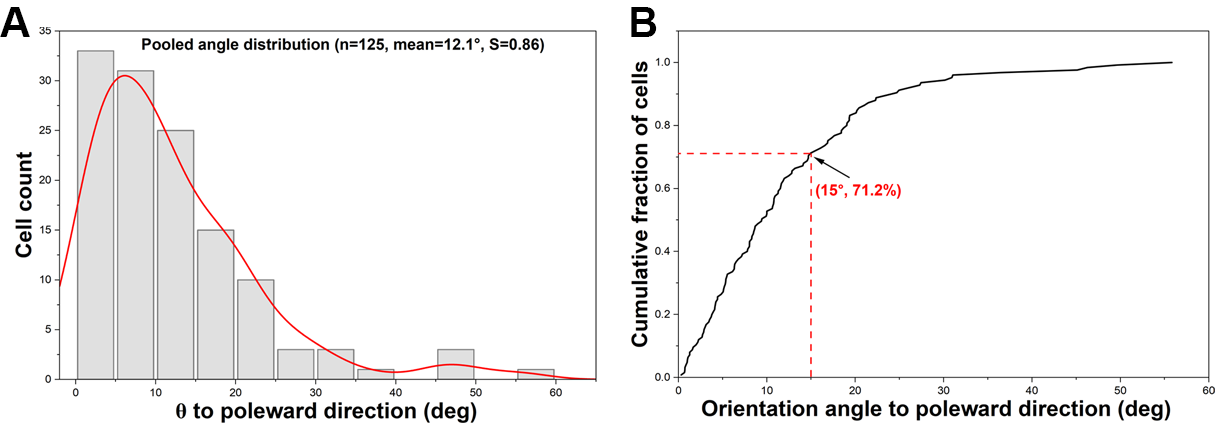


**Figure S3** Alignment analysis of C2C12 cells cultured on dcECM microspheres. (A) Histogram of pooled cell orientation angles (θ) relative to the poleward direction (Cell number = 125; bin width = 5°) with an overlaid kernel density estimation curve (Red). (B) Cumulative distribution function (CDF) of cell orientation angles; dashed red lines indicate the alignment threshold at θ = 15° and the corresponding cumulative fraction of cells (71.2%).
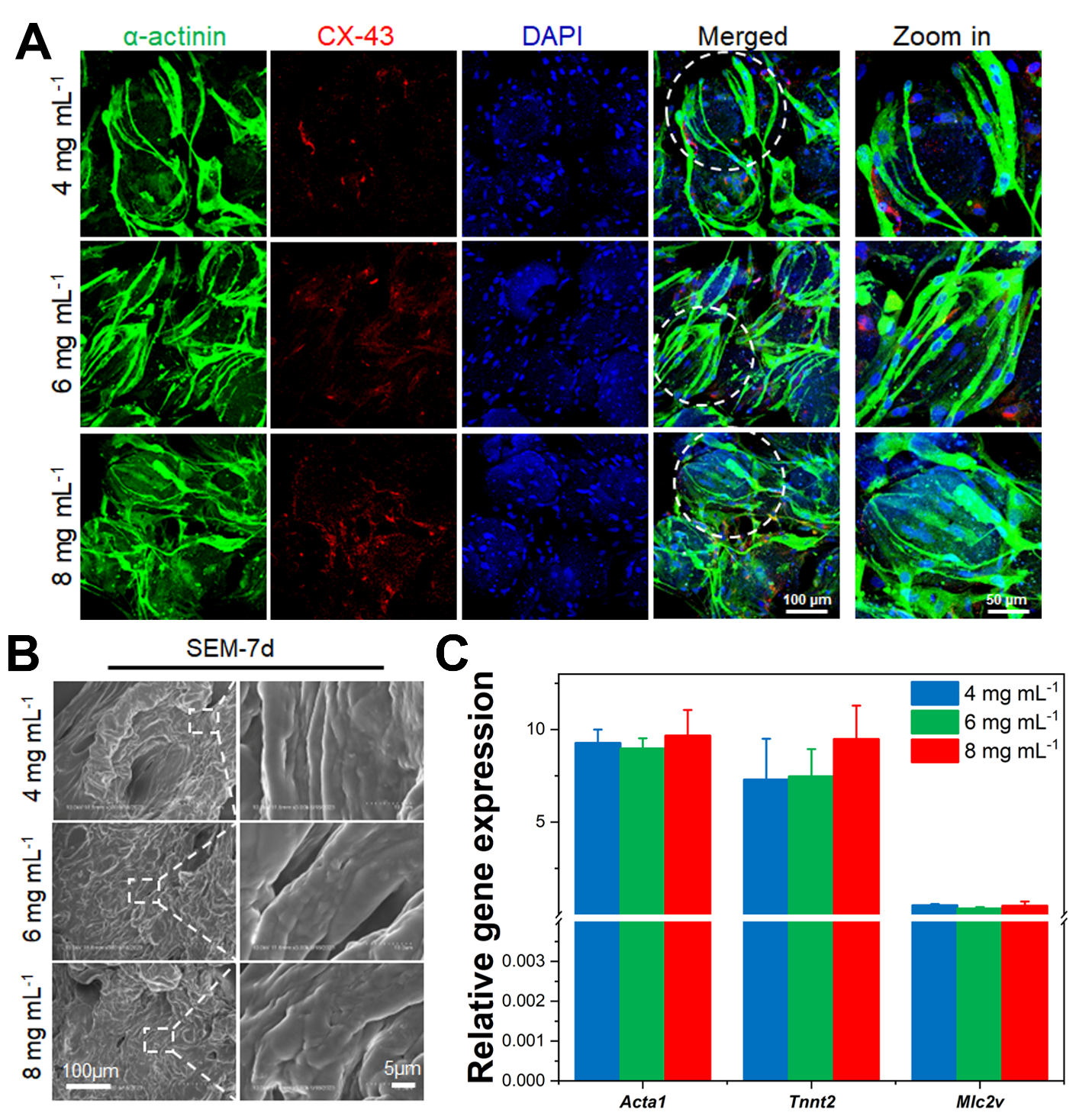


**Figure S4** Cardiac differentiation of H9c2 cells on dcECM microspheres with different concentrations. (A) Immunofluorescence staining of H9c2 on day 7 (green: α-actinin, red: CX-43, blue: DAPI). (B) SEM images of H9c2 cells on different dcECM microspheres at day 7. (C) Gene expression of H9c2 cell on different dcECM microspheres at day 7 (n=3). Data are expressed as mean ± SD. Analyzed by One-way ANOVA.

**Video S1** Calcium transient video of hiPSC-CMs cultured on TCP at day 14.

**Video S2** Calcium transient video of hiPSC-CMs cultured on 2D dcECM bulk hydrogel at day 14.

**Video S3** Calcium transient video of hiPSC-CMs cultured on 3D dcECM microspheres at day 14.

**Video S4** Brightfield video of spontaneous beating of hiPSC-CMs cultured on 3D dcECM microspheres at day 30.

**Video S5** Brightfield video of spontaneous beating of hiPSC-CMs cultured on 3D dcECM microspheres at day 60.

**Video S6** Brightfield video of spontaneous beating of hiPSC-CMs cultured on 3D dcECM microspheres at day 90.

**Video S7** Brightfield video of spontaneous beating of hiPSC-CMs cultured on 3D dcECM microspheres at day 120.

**Video S8** Brightfield video of spontaneous beating of hiPSC-CMs cultured on 3D dcECM microspheres at day 150.

**Video S9** Brightfield video of spontaneous beating of hiPSC-CMs cultured on 3D dcECM microspheres at day 180.

**Video S10** Brightfield video of spontaneous beating of hiPSC-CMs cultured on 3D dcECM microspheres at day 210.

**Video S11** Brightfield video of spontaneous beating of hiPSC-CMs cultured on 3D dcECM microspheres at day 240.
